# Following Camels Between Bone and Culture: Camel–Human Interactions in China from the Neolithic to the Late Imperial Period

**DOI:** 10.3390/ani16050772

**Published:** 2026-03-01

**Authors:** Yuxin Ding, Jiangsong Zhu, Jian Ma, Marcella Festa

**Affiliations:** 1 China–Central Asia “The Belt and Road” Joint Laboratory on Human and Environment Research, Northwest University, Xi’an 710069, China; 2 School of Cultural Heritage, Northwest University, Xi’an 710069, China

**Keywords:** camel, China, zooarchaeology, camel–human interactions, camel exploitation, camel dispersal

## Abstract

Bactrian camels (*Camelus bactrianus*) have long been recognized in China as key agents of long-distance connectivity, based largely on iconographic and textual evidence, while osteological data have rarely been incorporated into discussion. Because these data have seldom been examined within a unified analytical framework, current knowledge of the development and shifting patterns of camel–human relationships remains fragmentary. To address this gap, the present study provides a detailed analysis of available camel osteological material from archaeological contexts in northern China and integrates it with broader archaeological and historical evidence. Our results identify diverse forms of interaction across time and space, including camel exploitation for transport and labor, consumption, funerary practices, and craft production. Spatiotemporal patterns indicate a persistent concentration of osteological remains in China’s northern frontier zones, whereas the record remains sporadic in central regions despite increasing camel representations in material culture and texts. This enduring distribution reflects ecological suitability and sustained economic integration in arid zones. The absence of such conditions in Central China meant that camels were never fully incorporated into local everyday life; instead, they primarily operated within imperial logistical and political systems and came to be culturally important through their role in broader exchange networks.

## Simple Summary

Bactrian camels were key agents of long-distance interaction in China. Previous studies on camel-human dynamics have relied mainly on iconographic and textual data. This study integrates osteological material with broader archaeological and historical evidence to better understand long-term patterns of human–camel relationships. Results reveal diverse forms of interactions, including transport, labor, consumption, funerary practices, and craft production. Camel skeletal evidence consistently clusters in northern arid regions, where environmental conditions supported sustained economic use, whereas in Central China the record remains sparse despite increasing cultural representations. This distribution suggests limited practical integration in the everyday life of the Central Plains, with camels instead acquiring cultural significance through their role in wider exchange networks. These findings demonstrate how social demand and environmental conditions shaped camel use, offering new insight into early mobility, exchange and socio-cultural dynamics in China.

## 1. Introduction

The study of human–nonhuman relationships is a complex and still relatively underdeveloped area of archaeological research, yet it offers significant potential for understanding ancient societies, socio-economic transformations, and patterns of interaction [1,2,3,4,5,6,7]. One major turning point in human–animal dynamics was the process of domestication and the emergence of secondary product exploitation, which fundamentally reconfigured earlier relationships characteristic of hunter–gatherer systems [8,9]. Human–animal relationships can be approached through the diverse ways in which animals became incorporated into socio-economic systems, operating at different scales and with varying degrees of archaeological visibility [10,11,12]. Integration may be direct and physical, when animals are bred, managed, consumed, or otherwise incorporated in routine subsistence and production practices. It may also occur in more mediated or indirect forms, for example through their roles in state logistics, long-distance exchange, or specialized transport systems that do not necessarily leave a strong signature in local faunal assemblages. Finally, they may acquire primarily representational or symbolic significance within cultural and ideological frameworks, even where their everyday economic use remains limited. Investigating these different modes of integration is essential for developing a more nuanced understanding of the trajectories of human–animal relationships and their contribution to the socio-economic and ideological development of past societies.

The Bactrian camel (*Camelus bactrianus*), often referred to as the “ship of the desert,” became a key symbol of long-distance connectivity in China, as its exceptional ability to traverse Inner Asia’s vast desert and steppe landscapes historically made it indispensable for trans-regional transport and the movement of people, goods, and military forces [13,14,15]. As noted by [14], reliance on camels was economically efficient, even when compared to wheeled transport, as it did not require the construction and maintenance of complex infrastructural support systems. Beyond their role in interregional exchange, camels likely formed part of broader local economic systems, supporting mobility, subsistence, and pastoral lifeways, as suggested by modern practices in Mongolia, Kazakhstan, and northwestern China, where camel husbandry is still closely linked to rural livelihoods through transport, dairy production, and meat consumption [16,17,18,19]. Despite their historical importance, human–camel relationships in China remain relatively unevenly documented and poorly understood.

Drawing primarily on iconographic and textual sources, existing scholarship has mostly focused on the appearance of camels in the Central Plains during the Warring States (475–221 BCE) and Han (206 BCE–220 CE) periods, and associated it with growing interactions between populations in these regions and communities in the northwest [20,21,22,23]. A larger body of research on the Tang period (619–907 CE) has examined camel representations in material culture, in particular camel-shaped pottery figurines. Notably, some categories of cultural material used in these studies—particularly portable items—derive from poorly documented contexts and rely on broad spatial attribution and stylistic dating, which can introduce a degree of uncertainty in the interpretations (cf. Table S1). This body of work has tended to interpret camels mainly as indicators of contact with northern regions, emphasizing their role as symbols of Silk Road exchange [24,25,26]. Far less attention has been paid to other periods or to broader questions, including the modes and extent of their integration into regional socio-economic and cultural systems.

Addressing these issues requires the systematic use of osteoarcheological evidence, which has so far been only minimally incorporated into discussions (but see [27,28]). One reason for this limited attention likely lies in persistent uncertainties surrounding camel domestication and the difficulty of distinguishing wild from domestic forms. Unlike many herd animals, camel domestication did not involve intensive human control or a fundamental reorganization of the animal’s lifeways. Instead, particularly in desert transport contexts, it required humans to adapt to the camel’s ecological and behavioral constraints, blurring the boundary between wild and domestic populations [29,30]. Despite the availability of reference collections and identification manuals for camelids [31,32,33,34], their bones are notoriously difficult to identify at the species level, in particular when fragmentary, often resulting in misclassification or generic reporting of reduced analytical value [29,35]. These challenges are especially pronounced for older assemblages, studied before biomolecular approaches such as ZooMS or ancient DNA became available and often no longer amenable to reanalysis due to preservation conditions. Separating domestic from wild forms on osteological grounds alone remains problematic, with relatively confident attribution to domestication restricted to some osteometric parameters and securely dated finds outside the early to mid-Holocene distribution of wild camels [24,29]. Despite these limitations, camel skeletal remains in early archaeological contexts—whether wild or domestic—remain direct evidence of human–camel interactions. 

Another issue concerns the uneven geographical distribution of research on camels. Most zooarchaeological studies have focused on Western Asia [29,30,35,36], while other regions—most notably China—remain comparatively understudied (but see [37,38]). In China, archaeological and zooarchaeological research has traditionally prioritized sedentary agricultural communities in the Central Plains, particularly the Zhongyuan and Guanzhong regions [39,40,41], with the result that important contexts of camel–human interaction—often situated in desertic or semi-desertic landscapes—are relatively poorly represented in the archaeological record. Even within the Central Plains, zooarchaeology has largely focused on the domestication process and early exploitation of the *liu chu* (六畜, “six livestock”), often leaving other taxa, including camels, comparatively underexamined [40,41]. On a smaller scale, urban sites are affected by uneven recovery practices and preservation conditions. Chronological research bias against historical periods, when written sources become substantial, have also shaped the dataset [42,43]. Faunal recovery methods, sampling strategies, and reporting standards vary substantially among projects and over time, with more recent studies typically providing finer-grained identifications of anatomical elements, age profiles, pathological conditions, and human modifications (see [41] for a summary). Within this scenario, camel remains may have been underreported, misidentified, or not systematically collected. Furthermore, osteological evidence for camels is largely scattered across excavation reports and zooarchaeological studies and is often treated in isolation from remains documented elsewhere, as well as from broader bioarchaeological and cultural datasets.

As these materials have rarely been examined within a unified analytical framework, our understanding of the development and changing patterns of camel–human relationships remains fragmentary and uneven. In order to fill this gap, this study presents a detailed analysis of available camel osteological material from archaeological contexts in northern China and integrates these data with broader archaeological and historical evidence to (1) refine current interpretations of camel–human interaction and (2) reconstruct broad patterns of camel dispersal and integration across different regions of China.

### Camel Behavior and Domestication

The camel’s specific adaptation to desert environments has been extensively documented. Camels subsist on dry grasses, shrubs and halophytes, and can endure prolonged periods of food and water scarcity, due to a suite of physiological adaptations, including fat storage in the humps, highly concentrated urine, dry feces, and a high tolerance for fluctuations in body temperature [14,44,45,46,47].

The natural range of the extant wild two-humped camel (*Camelus ferus*) is largely restricted to the desert and semi-desert environments of Central Asia and northern China. Earlier scholarship regarded this species as the direct progenitor of the domestic Bactrian camel [29,48]. More recent genetic evidence, however, indicates a single domestication event from an unidentified, possibly extinct wild forms [49], with the giant camel (*Camelus knoblochi*) sometimes proposed as a potential candidate [50,51]. Some scholars argue that these animals were domesticated in Central Asia and later introduced into China [14,28,52], while others suggest northern China may have been one of the regions where this process occurred [53,54]. Although the exact location remains uncertain, the desertic landscapes of southern Kazakhstan, northwestern China and western Mongolia, overlapping with the present distribution of wild populations, remain the most plausible region for early camel domestication [55].

Unlike strongly gregarious herd animals such as sheep or cattle, camels are comparatively less social and more independent, forming small, loosely structured groups that move seasonally in response to the availability of pasture and water [56]. Camels are generally cautious and not naturally docile. In addition, they have slow life histories, with sexual maturity usually reached only at five years, gestation lasting approximately 390–410 days, and interbirth intervals often exceeding three years [37]. Although these traits may have complicated early domestication, several factors—including periods of climatic stress and species-specific behaviors such as strong spatial memory, which promotes repeated use of calving areas and reliable grazing zones—are believed to have encouraged recurrent interactions with humans and facilitated the initial taming of camels [30,57,58]. The specific pathways leading to domestication remain, however, poorly understood.

The reason for camels’ early management is also largely speculative [14,35,58]. Scholars suggest that they were first exploited for meat and hides, although their suitability as load-bearing animals would have likely been recognized early on [59]. Following domestication, camels have proved highly versatile and a key resource in arid and semi-arid environments, where they can be trained to carry heavy burdens, while also providing milk, meat, and fiber [16,17,18,60].

## 2. Materials and Methods

Osteological data were compiled between 2024 and 2025 through a structured review of zooarchaeological studies, excavation reports, and other publications reporting camel remains, targeted searches in the CNKI database (https://www.cnki.net (last accessed on 20 January 2026), library research at Northwest University, and direct communication with specialists. For the assemblage from K15 at Heishanling, the complete faunal assemblage was examined directly by the authors [61]. For each occurrence, standardized metadata were recorded, including site and context, location, chronological attribution, and skeletal element and count. Evidence of anthropogenic surface modification and pathological changes was also documented and assessed. The level of zooarchaeological detail is uneven across sources, with some publications, particularly excavation reports, not always providing complete information on skeletal elements present, age profiles, metric data, or other key zooarchaeological variables. Records reported only in generic terms (e.g., *Camelus* sp.) or with uncertain specimen types and counts were flagged and their interpretative weight treated accordingly. With few exceptions [37], most camel bones have not been directly radiocarbon dated, and their chronological attribution therefore relies on the dating of the archaeological contexts in which they were recovered.

The dataset spans a broad chronological range from the Neolithic to the late imperial period, allowing a long-term assessment of human–camel interactions. For analytical comparability, occurrences were grouped into four major stages—Neolithic–Bronze Age (ca. 3000–1000 BCE), Iron Age (ca. 1000–200 BCE), Han period (206 BCE–220 CE), and post-Han (after 220 CE)—which correspond to officially recognized historical phases in Chinese archaeology and historiography and are commonly employed in relevant analyses, thereby facilitating the identification of broad-scale diachronic patterns (Table 1). Although the post-Han category encompasses a long-time span, this grouping was adopted in light of the well-known scarcity of zooarchaeological research for medieval and late imperial China [43,62,63]. Combining these periods provides a sufficient body of evidence for meaningful analysis. Finer sub-periods within these categories are discussed where the evidence allows. Although “camel-like” fossils and remains attributed to the extinct giant camel are known from Pleistocene contexts in northern China [41,64], the Paleolithic period is excluded from this study, as such materials are typically examined within paleontological and paleoanthropological frameworks rather than zooarchaeology.

Faunal records for each period were systematically cross-referenced with non-osteological evidence, including iconographic materials (primarily rock art, camel-shaped objects, mural paintings, and when available, representations on textiles) and written sources (mainly official histories and excavated administrative documents). Given that some categories of material—especially portable objects such as figurines and camel-shaped artifacts—may derive from poorly documented contexts, the analysis prioritized, where possible, well-contextualized and securely studied specimens with reliable chronological attribution. This triangulation was used to assess camel presence, contextualize probable forms of camel–human interaction and identify convergences or discrepancies between skeletal, visual, and textual records in order to evaluate diverse modes of camel dispersal.

Osteological occurrences and selected iconographic data points (Table S1) were georeferenced and mapped in a GIS environment to investigate spatial and temporal patterns of camel distribution in China. Due to the high density of post-Han cultural materials, which would obscure earlier spatial patterns, these data are presented separately in Table S1 and Figures S1 and S2 and are discussed in the main text together with the relevant references.

## 3. Results

### 3.1. Osteological Evidence

**Figure 1:**
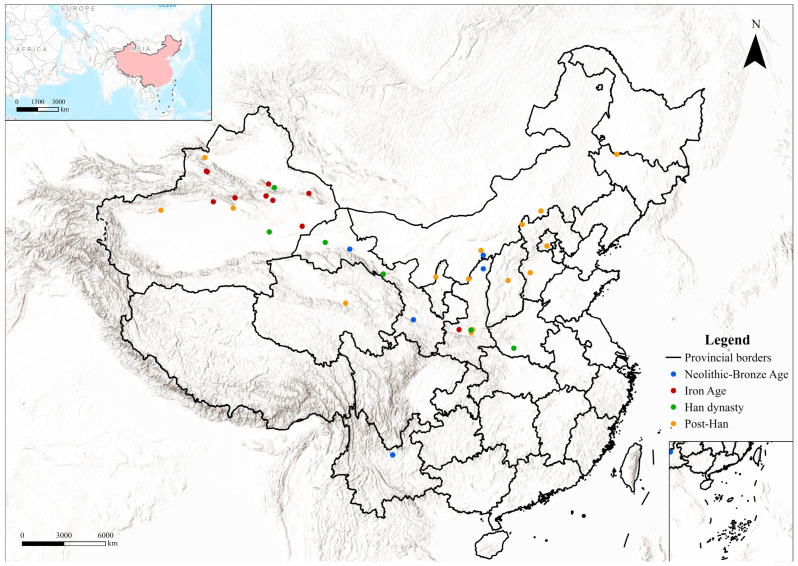
Distribution of osteological evidence in China by period. The map was created using ArcGIS Pro 3.0.2 (Esri; https://www.esri.com/en-us/arcgis/products/arcgis-pro/overview; last accessed on 19 January 2026.). The basemap is the built-in Esri “World Topographic Map” (©2022 Esri Inc, 380 New York Street, Redlands, CA 92373, USA). Administrative boundaries were obtained from the Tianditu Cloud Center administrative division dataset (© Tianditu Cloud Center; managed by the National Geomatics Center of China under the Ministry of Natural Resources, Beijing, China; accessed on 13 January 2026; available at https://cloudcenter.tianditu.gov.cn/administrativeDivision).

Currently, 39 archaeological sites in Northern China have been reported to contain camel bones (Table 2; Figure 1).

**Figure 2:**
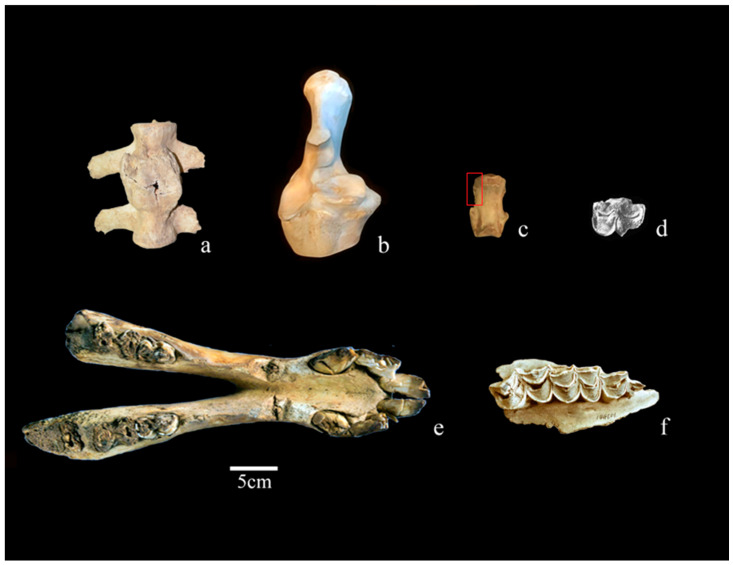
Selected camel osteological evidence from China: (**a**) Deformed lumbar vertebra from Shichengzi (after [78]); (**b**) Radius and ulna from Xitucheng identified as bone working waste (after [96]); (**c**) Second phalanx with osteophytes (red square) from Dalete (after [94]); (**d**) Upper molar from Zhukaigou (after [67]); (**e**) Mandible with cut marks in Tongwancheng (after [87]); (**f**) Maxilla from the sand pit along the Weihe River, Gaoling (after [89]).

The earliest camel remains currently documented from archaeological contexts in China lack secure domestication status and include an upper molar from Phase IV at Zhukaigou, in the Ordos region of Inner Mongolia [67] (Figure 2d), as well as a left maxilla with P3–M3 and a left metatarsal from Muzhuzhuliang in Shenmu, northern Shaanxi [66], both dating to the late third to early second millennium BCE. Additional early material has been reported from Huoshaogou in Yumen, Gansu, however, its chronological attribution remains uncertain, as different publications associate the remains with Neolithic and Iron Age contexts, without clarification as to whether they represent the same material or distinct finds [68,69]. Single camel bones of comparable antiquity have been reported from the Siwa site in the upper Beiluo River region of Shaanxi and from Wuding Xincun, in Yunnan, respectively, but the available contextual information is limited and the finds remain taxonomically ambiguous given the limitations of the published evidence [63,65].

More substantial camel assemblages are documented at sites dating to the first millennium BCE. While the domestication status of these specimens is often inferred from contextual evidence [29], some have been more securely assigned to Bactrian camels through morphology and biometry [24]. At the cemeteries of Qunbake (Luntai) and Jialekesikayinte (Nilka) in present-day Xinjiang, camel heads—interpreted as deriving from domestic animals on contextual grounds—were recovered from several graves [72,75]. At Shirenzigou, 158 skeletal fragments excavated from tomb M12 were securely attributed to a single 7–8-year-old Bactrian camel on the basis of detailed morphological analysis [37]. Additional five bones were recovered from overlying architectural remains and domestic contexts at the site. The assemblage was examined for cut marks and pathological alterations. At the Heishanling mining site K15, morphological and contextual analysis has currently identified a dozen skeletal elements of domestic camel—including ribs, phalanges and cranial bones [61]. Anthropogenic and pathological modifications affecting these remains have been assessed.

The Pingling burial pit, associated with Emperor Zhao of Han (87–74 BC), yielded the remains of 33 camels, but methodological constraints linked to site conservation prevented other detailed zooarchaeological analysis beyond species identification [83,84]. Additional camel bones and teeth have been reported from the Xinmang-period (9–23 CE) coin-casting workshop in Nanyang, but accurate contextual information is limited [85]. Further northwest, in Baguaying cemetery, at Zhangye, Gansu, some ribs and scapulae were recovered from grave M35, while at Goubei in Turfan, four camel sacrificial pits contained complete animals. Tomb M16 alone yielded three camels, while M01 and M06 each contained a single individual [74]. Additional funerary camel remains from the Han period come from Sangeqiao in the Ili region of northwestern Xinjiang [76]. At Yuansha ancient city (Xinjiang)—where the city wall is radiocarbon-dated to around 200 BCE—531 domestic camel skeletal fragments represented over 20% of the faunal assemblage [80]. From the same region, camel bones from Sanjianfang in Loulan bear cut marks and chopping traces [79]. A total of 17 skeletal fragments, including pelvis and lumbar vertebrae, some exhibiting lesions, were recovered from the Shichengzi military fort in Changji [78] (Figure 2a). These findings have been identified as *Camelus* sp., although contextual information strongly suggests these were domestic specimens [38].

Post-Han material comprises eight domestic camel bones—including cranial elements and long bones—from Tongwancheng at Jingbian (Shaanxi), dating to the Eastern Jin period (317–420 CE) [87]. These specimens were examined for anthropogenic and pathological modifications (Figure 2e). Tang-period evidence consists of five elements of domestic individuals from the West Market workshop of Chang’an (Xi’an) bearing traces of working processes [91], as well as a fragmentary right maxilla with P3–M2 from a sand pit along the Weihe River near Gaoling (Xi’an) [89] (Figure 2f). A single camel first phalanx is reported from Jinyang Ancient City in present-day Shanxi [90]. Funerary contexts of the same periods including Bactrian camel bones are the Harisai cemetery in Dulan, Qinghai, where four individuals had been sacrificed in a single pit [92]. Later evidence comes from the shaft of tomb M07 in the Western Xia cemetery at Minningcun, Yinchuan, Ningxia (1038–1227 CE), where 24 bones from a single individual were identified as domestic Bactrian camel through osteometric analysis of teeth and metacarpals [24]. Additional finds dating to the Jin period (1115–1234 CE) are 31 bones from the Xitucheng site in Kangbao, Hebei—22 of which were identified as worked elements [96] (Figure 2b)—as well as two fragments from Guangyuanli, in Beijing [38]. One camel calcaneus and two sesamoids were excavated from Daqingshan in Zhaoyuan, Heilongjiang [95]. Late imperial evidence for camels is documented at Yanjialiang, a Yuan-period rural settlement in Inner Mongolia (1271–1368 CE) where 44 Bactrian camel bones were recorded and measured [97]. The roughly contemporaneous Xiguanxiang site, also in Inner Mongolia, contained 22 bones, mostly scapulae and metapodials [98]. At the Dalete ancient city in Xinjiang, 155 Bactrian camel skeletal fragments from at least eight individuals were identified and pathological marks examined in detail [94] (Figure 2c). In Zhengding, Hebei, ten domestic camel bones were recovered from the southern plaza of Kaiyuan Temple, nine of which bear regular saw marks [38]. At the Qing-period Yilin Post Station site in Erenhot, Inner Mongolia, 109 camel bones were excavated from ash pits and cultural layers [99].

### 3.2. Non-Osteological Evidence

Beyond osteological remains, other bioarchaeological indicators of camels are rare and often equivocal. Reports of domestic camel dung from Talitaliha in Dulan County, Qinghai, dated to ca. 900 BCE [100,101], cannot be independently verified [28]. Similar uncertainty applies to dung remains reported from Yingyayilake in Tuokexu County, Xinjiang, attributed to the Han period [102]. Camel-derived textiles and fibers are documented at a limited number of Iron Age sites, primarily in Xinjiang [2,103,104].

**Figure 3:**
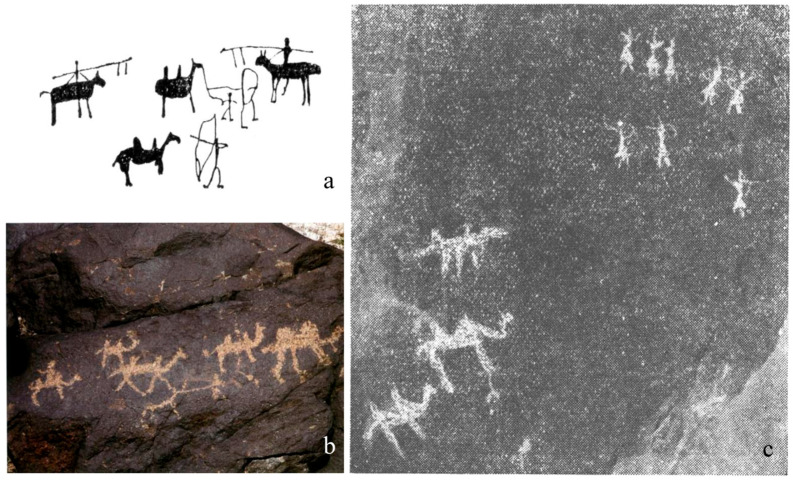
Rock art in China (selection): (**a**) Hutubi (Iron Age, Xinjiang) (after [107]); (**b**) Alashan (Iron Age, Inner Mongolia) (after [107]); (**c**) Heishan (prehistory, Gansu) (after [105]).

Other forms of archaeological evidence are more widespread, with rock art mainly from northern regions, ancient texts largely produced in Central China, and figurative materials (decorated items, camel-shaped objects and mural art) occurring across much broader areas. The earliest camel depictions occur in Neolithic and Early Bronze Age rock carvings across present-day Inner Mongolia, Gansu, and Xinjiang. They have been dated on the basis of stylistic and contextual criteria and portray various forms of human–camel interactions, including hunting and herding [64,105,106] (Figure 3). Rock art production in these regions continued into later periods, extending as far as the Yuan (1261–1368 CE) and Ming (1368–1644 CE) dynasties, though these phases remain comparatively understudied [107].

In written sources, camels appear from the late first millennium BCE onward, most commonly referred to as *tuotuo* (橐驼), but also as *pangniu* (牥牛) and *fengniu* (封牛) [28,64]. References occur in texts such as the *Shanhaijing* (山海经 Classic of Mountains and Seas), *Yi Zhou Shu* (逸周书 The Lost Book of Zhou) and *Mu Tianzi Zhuan* (穆天子传 The Travels of King Mu), where camels are frequently associated with long-distance travel, desert crossings, and western remote regions, sometimes in mythic or semi-legendary contexts [28,64]. During the Han period, camels are mentioned more realistically in historical sources, most notably in the *Shiji* (史记 Records of the Grand Historian), particularly in the *Xiongnu Liezhuan* (匈奴列传 Account of the Xiongnu), where they are mostly linked to northern frontier groups active during the consolidation of early dynastic states. Further evidence is provided by administrative documents and official histories, including bamboo slips excavated from Xuanquan Station near Dunhuang [108], and accounts in the *Han Shu* (汉书 Book of Han), especially the chapter on the Western Regions (西域传 *Xiyu Zhuan*) [97,98]. In Tang-period official histories, including the *Jiu Tang Shu* (旧唐书 Old Book of Tang) and the *Xin Tang Shu* (新唐书New Book of Tang), camels appear within geographical descriptions and accounts of frontier regions and foreign polities [64]. Administrative records, such as the *Tulufan Chutu Wenshu* (吐鲁番出土文书 The Turfan Documents) provide concrete evidence for the involvement of these animals in transport and trade [109]. In texts composed after the Tang period, references to camels remain relatively frequent in the dynastic histories of the Liao (907–1125), Song (960–1279), Jin, and Yuan, where they are still associated with frontier economies, long-distance transport, and state logistics.

**Figure 4:**
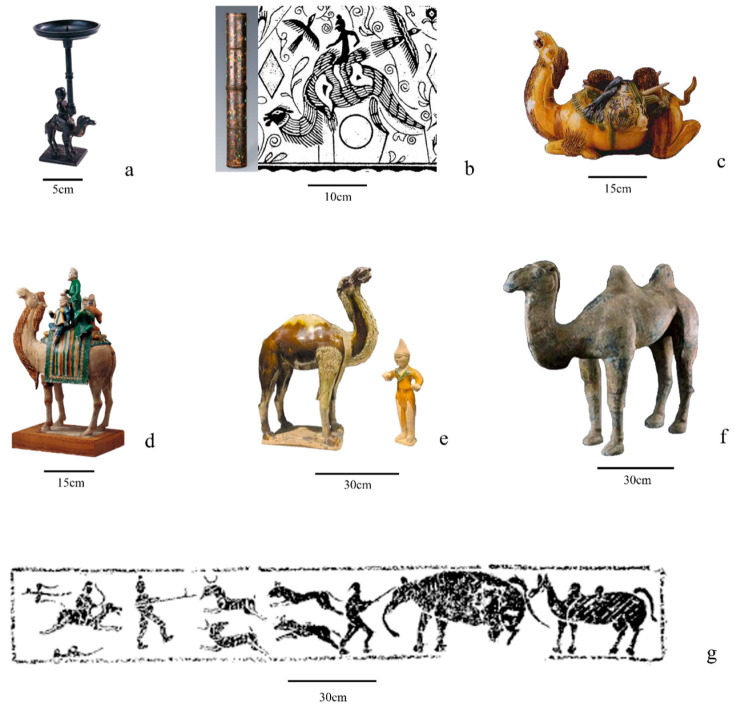
Cultural representations of camels in China: (**a**) Bronze camel-rider lamp from tomb M2 at Wangshan, Jiangling, Hubei (Warring States) (after [111]); (**b**) Gilt- and silver-inlaid bronze chariot parasol handle ring decorated with camel motifs from tomb M122 at Sanpanshan, Ding, Hebei (Western Han) (after [112]); (**c**) *Sancai*-glazed earthenware camel shown kneeling and carrying goods, from Tomb 31 at Nanxiao, Xi’an (Tang) (after [113]); (**d**) *Sancai*-glazed earthenware camel carrying musicians, from the tomb of Xianyu Tinghui, General of Yunhui (723 CE), Xi’an (Tang, National Museum of China, Beijing); (**e**) *Sancai*-glazed earthenware one-humped camel led by a Hu attendant (Tang, Xianyang Ancient Ferry Site Museum); (**f**) Pottery camel from a tomb at Nanxiao, Xi’an (Western Han; Xi’an Museum); (**g**) Mural carving depicting a hunting scene with camels from Zoucheng, southern Shandong (Han) (after [114]).

**Figure 5:**
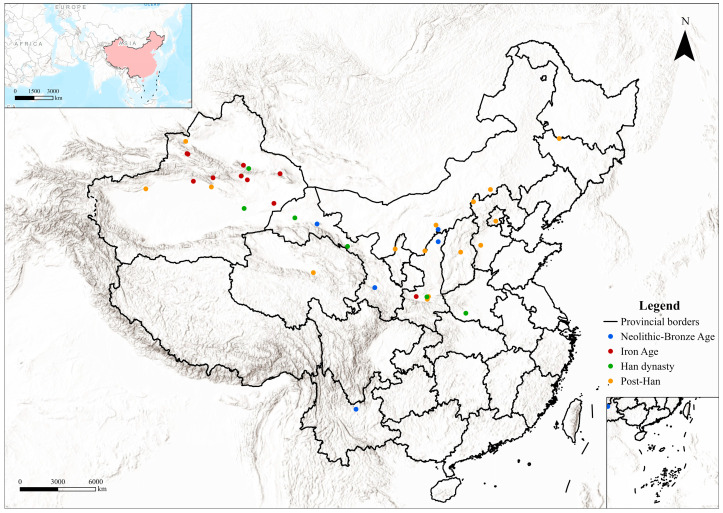
Distribution of camel-related cultural materials across three chronological stages: NB (blue), IA (red), H (green) and PH (only rock art in yellow). The distribution of all cultural material for the phase PH can be found in Figure S1. Detailed information on the cultural material can be found in Table S1. The map was created using ArcGIS Pro 3.0.2 (Esri; https://www.esri.com/en-us/arcgis/products/arcgis-pro/overview; last accessed on 19 January 2026). The basemap is the built-in Esri “World Topographic Map” (©2022 Esri Inc, 380 New York Street, Redlands, CA 92373, USA). Administrative boundaries were obtained from the Tianditu Cloud Center administrative division dataset (© Tianditu Cloud Center; managed by the National Geomatics Center of China under the Ministry of Natural Resources, Beijing, China; accessed on 13 January 2026; available at https://cloudcenter.tianditu.gov.cn/administrativeDivision).

Figurative representations in the form of camel-decorated and camel-shaped objects first appeared in Central China by the mid-first millennium BCE (Figure 4a) and became increasingly common during the Han period, with both their number and diversity expanding to include figurines, murals, decorated objects and textiles [22,110] (Figure 4b,f,g). The peak of Silk Road exchange in China occurred during the Tang period, which produced the largest corpus and the most elaborate camel figurines, most notably *sancai*-glazed examples [20,21] (Figure 4c,d). In northern regions, such representations also occur in each period, though less frequently (Figure 5).

## 4. Discussion

### 4.1. Camel–Human Interactions and Practices

The integration of osteological, other bioarchaeological and cultural evidence reveals various forms of human–camel interaction and multiple uses of camels across time and space.

#### 4.1.1. Transport and Labor

From a zooarchaeological perspective, transport and labor activities can be inferred through pathological changes in the animal’s skeleton. Load-bearing stress may manifest in enlargements of articular surfaces or other structural deformations of foot elements [115,116] and vertebral pathologies—such as osteophyte formation or vertebral fusion. Such indicators have been more extensively studied in cattle and horses [1,115,117,118,119,120], while research on camels remains more limited [121]. In addition to skeletal markers, contextual data can also be informative [122,123,124]. For example, the recovery of camel remains from settings such as markets, postal or relay stations, or other transport-related facilities may provide support for their logistical roles.

Osteological evidence for camel use in transport during the Neolithic and Bronze Age is absent. This may partly reflect the fragmentary nature of the assemblages and the poor preservation of diagnostically informative skeletal elements. Iconographic material from the Gobi Desert and Xinjiang depicts camels primarily in hunting contexts, within herds, or occasionally under human control, indicating early human–camel interactions but offering no clear evidence for riding or load-bearing use [64,105,107] (Figure 3c). Overall, the available information suggests that such uses were likely not yet widespread at this time.

Skeletal indicators of camel labor use appear in the first millennium BCE in Northwest China. At Shirenzigou, pathological changes have been observed on the 11th and 12th thoracic vertebrae of the individual buried in tomb M12, including osteophyte formation and incipient fusion, together with bony outgrowths on a third phalanx [37]. Rock art from Inner Mongolia and Xinjiang depicting mounted camels further supports their exploitation for riding or load-bearing during this period (Figure 3b). These developments broadly coincide with the intensification and expansion of pastoral systems, the development of horseback riding, and increased regional mobility [1,2,120]. Within this context, the adoption of camels as transport animals would have offered clear advantages for managing movement and exchange across increasingly large and environmentally diverse landscapes of Northwest China.

Thers is no direct zooarchaeological evidence for such uses outside these northern regions, however, Iron Age camel-shaped objects from Central China showing mounted riders indicate that camels were somehow conceptually understood as animals capable of carrying people or loads [111,125] (Figure 4a). During much of the first millennium BCE, contacts between the Central Plains and the arid northwest were likely intermittent and mediated through frontier groups rather than supported by stable state logistics [126,127,128]. Under these conditions, demand for regular desert transport in the Central Plains would have been limited. Given the camel’s specialization for arid long-distance movement, its routine use in the more humid agricultural core was likely unnecessary, which accounts for the rarity of camel remains in the region.

Vertebral anomalies in camel remains from the Han-period Shichengzi military fort in Xinjiang have been interpreted as evidence of repetitive stress associated with load bearing labor, possibly connected to regional military provisioning [78,129] (Figure 2a), supporting the continued use of camels in local transport systems. From the same region, a woolen skirt from the Shanpula cemetery depicts camels draped with cloths resembling saddle blankets or load covers, suggesting riding or pack use [110]. No pathological evidence for camel labor has been documented in Central China. Depictions of camels being ridden or carrying loads on figurines, murals and other materials, however, increases significantly. In the Pingling burial pit in Xianyang, for example, were discovered painted wooden models of two-humped camels pulling carts [83]. A gilt silver–inlaid bronze chariot parasol ring handle excavated from grave M122 at Sanpanshan, Ding County, Hebei, was decorated with an image of a ridden camel [130] (Figure 4b). The apparent scarcity of pathological evidence for camel labor in the Central Plains may partly reflect uneven research intensity. Notably, the large deposit of 33 camels from the joint burial pit of Emperor Zhao of Han has not been systematically examined for transport-related stress indicators due to site conservation limitations, leaving their functional histories unresolved. However, the regional contrast in camel use during the Han period likely also relates to structural and ecological factors. Following the establishment of the Western Regions Protectorate (60 BCE), long-distance connectivity became more structured. As a result, camels likely played an increasingly important role in northwestern transport systems, as shown at sites such as Shichengzi in Xinjiang [78,129]. Within the relay-based organization of Han transport networks [128], however, camels would have primarily been deployed in these arid frontier zones and only occasionally reached the Central Plains, explaining the more limited archaeological record observed in Central China.

After the Han period, osteological evidence for camel use in transport remains limited, but a notable case comes from the ancient city of Dalete in Xinjiang, where camel remains show load-related stress marks [94] (Figure 2c). The recurring recovery of camel bones from contexts associated with exchange and mobility, such as postal or relay stations and urban markets, further supports their continued role in logistical networks. No cases of labor-related pathologies have been reported for camels in Central China. This pattern may partly result from research bias and the limited number of well-documented specimens from the region, many of which are either insufficiently specified or derive from contexts, such as market workshops [91] where transport-related pathologies are not expected to be detected. At the same time, the contextual association of these remains with exchange-related settings, such as urban commercial areas, is consistent with their use in logistical activities (Table 2). Figurative depictions of camels carrying goods and people become increasingly common across northern China, especially in the Central Plains during the Tang dynasty (e.g., [20]) (Figure 4c,d), coinciding with the peak of Silk Road exchange networks and supporting the growing importance of these animals in long-distance mobility. The use of camels as burden animals in northern China continued into later historical periods, up to the Qing dynasty, when they were still employed in coal transport from the Western Hills to the urban center of Beijing [131].

#### 4.1.2. Food

Most scholars agree that initial human engagement with camels was driven largely by meat exploitation [30,35,59]. Neolithic and Bronze Age rock art is dominated by hunting scenes [64,105,107,132] (Figure 3c). Although direct zooarchaeological evidence of meat consumption, such as butchery marks or marrow-processing breakage is absent [133], camel bones and teeth from early sites of Zhukaigou, Muzhuzuliang, and Huoshaogou are believed to have derived from wild or domestic individuals hunted for food [37,38,66,67,68].

Camel meat consumption is more clearly documented from the Iron Age in Xinjiang, where cut marks and breakage associated with marrow retrieval have been observed on bones from Shirenzigou [37]. During the Han period, butchery practices have been identified at Shichengzi and Yuansha [38,78,129]. After that, evidence of meat processing extends across both northern and more central regions, including butchered skeletal elements from Sanjianfang [79] and Tongwancheng Xicheng (Yulin, Shaanxi) [87] (Figure 2e). In later historical contexts, large quantities of camel bones recovered from ash pits and occupation layers at the Qing-period Yilin Post Station in Inner Mongolia have been associated with meat consumption [99].

Overall, evidence for camel exploitation for meat in China remains limited and poorly contextualized, preventing firm assessment of the nature of consumption, whether quotidian or ritualized—although the available data leave open the possibility that both occurred in different contexts, as briefly outlined below. The limited faunal signal partly derives from research biases that have focused primarily on species identification rather than broader subsistence practices [134]. These patterns should also be considered in light of factors like camels’ slow reproduction rates and the substantial time and skill required for training, which likely increased their value as transport animals and reduced their primary use for meat [135]. Ethnographic evidence from Xinjiang, Mongolia and Kazakhstan indicates that even today camels are principally used for transport, and are sometimes slaughtered for food only at an advanced age after the end of their working life [16,17,18,19]. Within a broader animal-economy framework, camels in northern China appear to have functioned mainly as high-value logistical assets rather than as regular meat resources. In the Central Plains, the rarity of evidence for camel consumption may additionally relate to the generally low archaeological visibility of the species in this region, which makes such practices statistically difficult to detect. Cultural factors may also have contributed to this pattern. As animals not traditionally integrated into established livestock regimes, but often associated with frontier or foreign contexts, camels were likely not routinely incorporated into local dietary practices.

#### 4.1.3. Funerary Sacrifice

**Figure 6:**
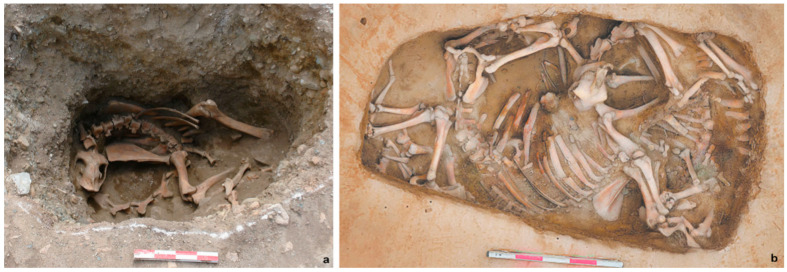
Camel burials. (**a**) Shirenzigou (M12K1) (after [37]) and (**b**) Harisai (after [92]), both in present-day Xinjiang.

Camels appear in mortuary settings as sacrifices or offerings, albeit less frequently than other animals [41]. Up to the Iron Age, such practices were confined to northern areas of present-day Xinjiang, where different funerary treatments are documented at cemeteries such as Qunbake, Jialekesikayinte, Shirenzigou, and Guobei [37,72,74,75] (Figure 6a). The placement of camel heads at Qunbake and Jialekesikayinte fits well within the broader “heads and hooves” tradition widely observed in pastoral burials across Eastern Eurasia during the second–first millennia BCE, and often intersected with practices of commensality and feasting [4,5,136,137]. Complete camel individuals are documented at Shirenzigou and Guobei. Notably, the Shirenzigou camel was relatively young, 7–8 years old, well below the species’ potential lifespan of over 30 years. While its early death has been linked to excessive load stress from riding or transport [41], intentional selection cannot be excluded. The sacrifice of young and potentially valuable transport animals may itself have functioned as a display of wealth and social capacity, particularly in the context of emerging local elites, which is the case of Shirenzigou and more broadly the Tianshan region during this period [1,138,139,140,141]. Although the inclusion of camels indicates that they had entered the symbolic and ritual sphere of pastoral communities, their participation remained selective, and comparable funerary practices more commonly involved horses, caprines, and cattle. The lower frequency of camels may partly result from recovery and identification biases, but cultural and economic factors were likely also important. In particular, as high-investment animals with specialized logistical value in arid mobility systems, camels were probably less readily diverted into routine funerary sacrifice than more reproductively flexible and locally abundant livestock.

Although the tradition of burying camels extended into Central China during the Han period—most notably with the interment of 33 individuals in the Pingling burial pit [83]—direct use in rituals and funerary contests remained prevalent in northern regions. This pattern continued in later periods, with camel burials remaining sporadic in Central China despite increasing associated cultural materials (Figure 4c,d,f,g), in comparison with northern regions (e.g., at Harisai and Reshui in Qinghai and Minningcun in Ningxia) [24,92,93] (Figure 6b). This distribution may partly result from research bias, but cultural factors could also have also played a role. Even where the broader economic and cultural significance of camels was recognized, these animals did not form part of the agriculture-based socio-economic and cultural traditions of the Central Plains and may not have been routinely incorporated into established burial practices.

#### 4.1.4. Production

Beyond their use for transport and meat, camels can provide a range of secondary products, including milk, hides, fiber, and bone [16,17,18]. Although it is reasonable to assume that early hunters and herders made broad use of camel carcasses, direct archaeological evidence for these practices remains limited, partly due to earlier research frameworks, which were not strongly anthropologically oriented [134], and to methodological constraints, as some relevant analytical techniques have only been developed or consistently used relatively recently. To date, no zooarchaeological or biomolecular analyses have directly demonstrated historical camel milk exploitation in China. Nevertheless, such use is plausible. Camel milk can be processed into durable products such as yogurt, well suited to mobile and long-distance lifeways [142]. Ethnographic evidence from Mongolia and northwestern China shows that camel dairy products remain highly valued, with certain breeds specifically maintained for milk production [143].

Camel fiber exploitation is better documented archaeologically, although existing studies remain limited in number and are largely confined to Iron Age contexts in present-day Xinjiang. This pattern likely reflects both the increasing research interest in the early pastoral economies in this region [1,2,3,120,144], and preservation biases, as the arid environments of Xinjiang favor the survival of textile materials. A textile fragment from a mid–first millennium BCE tomb at Alagou (Turfan) was woven using camel hair as the warp and sheep wool as the weft [103] and at least 42% of the textile pieces from the roughly contemporaneous Jirzankale cemetery (Tashkurgan) were made from mixed camel and sheep fibers as well as of pure camel underhair [2]. Additional evidence derives from the Iron Age site of Djoumboulak Koum in the Keriya River valley [104].

Bones were another important resource from camels which could have been used as raw material for tool production. Evidence for this practice in early periods is limited., however, without biomolecular methods such as ZooMS or aDNA, the possibility of selective species use for heavily worked elements must be considered, as intensive modification can obscure diagnostic features and create bias against the identification of camel in bone tool assemblages. At Shirenzigou, for example, despite the assemblage having been extensively studied, no worked bone has been securely identified as camel, but some non-diagnostic fragments may have been included within the general “large mammal” category [3,145]. Clearer evidence appears in later phases. At the Tang-period bone workshop in the West Market of Chang’an (Xi’an), at least five camel bones were identified among the raw materials [91]. At the Jin-dynasty Xitucheng site, 22 camel bones, including radii, tibiae, metacarpals and metatarsals, have been associated with bone-working practices [96] (Figure 2b). At the Kaiyuan Temple site in Zhengding, nine of ten camel bones, mainly metapodials, bear regular saw marks indicative of processing activities [38].

### 4.2. Distribution of Camels in China: Animals and Imagery 

In China, the earliest zooarchaeological evidence for camels—whose domestication status remains uncertain—closely follows their ecological preference for arid and semi-arid environments in the Gobi Desert and the adjacent Ordos Plateau [64,146,147]. Neolithic–Bronze Age osteological remains from Zhukaigou, Muzhuzhuliang, and Huoshaogou [37,66,67] and rock art are concentrated in these areas [64,105,148]. The limited skeletal evidence from China’s far northwest—modern Xinjiang—has been taken to suggest an earlier presence, and possible domestication, in what are now Inner Mongolia and Gansu [38]. However, Neolithic and Bronze Age rock art depicting camels is widely distributed across the Tianshan and Altai regions [106,107] and given the well-known scarcity of excavations from this period in this part of China [149,150], the apparent lack of osteological material may well reflect research bias rather than actual absence.

An early Bronze Age camel astragalus from Xincun in Wuding, Yunnan [65] lies outside the currently documented ecological and cultural range of camels and lack independent taxonomic confirmation. Although the presence of camels—wild or domestic—in southern China at such an early date is unlikely, it cannot be entirely ruled out. Some camel remains and Bactrian camel figurines dating to the late third and early second millennium BCE are known from the Indus Valley and adjacent Baluchistan [29,151,152,153]. Prehistoric interaction networks linking Yunnan with surrounding regions, including Southeast Asia, Indus and northern China [154,155] could in principle account for such occurrence. However, for the moment this remains a controversial and isolated finds that requires further corroboration and is not indicative of sustained camel presence in southwest China.

Current evidence indicates an increased presence of camels in Northwest China from the Iron Age, in the early first millennium BCE (Figure 1). Morphological and biometrical analyses of selected specimens from this region confirm the presence of domestic individuals (e.g., [37]), but in some cases domestic status has been inferred primarily from archaeological context (see [38] for discussion). This pattern at least partly derives from the uneven development of zooarchaeological research in the region. At sites such as Qunbake and Jialekesikayinte, for example, the available sources consist of excavation reports that mention “camels” and implicitly treat them as domestic based on contextual associations, but without providing detailed zooarchaeological assessment. Our results indicate that at this time camels served multiple roles within local societies, contributing to transport, subsistence, ritual practices, and likely craft production, and became integral to the regional socio-economic landscape. Rock art from this period increasingly depicts camel herds and riding scenes [106,107]. One particular panel from Hutubi, in Changji (Xinjiang) shows two mounted hunters with spears and bows intruding aggressively, apparently contesting camel grazing land—further supporting closer human–camel relationships in this region [107] (Figure 3a).

Textual sources produced in Central China during the Iron Age also document the growing importance of camels as transport animals in the northwest, while also pointing to increasing awareness of this species among Central Plains populations. The term *tuotuo* (橐驼), one of the ancient designations for camels, appears in bronze inscriptions from the later Zhou period (771–256 BCE), and the *Yi Zhou Shu* records their import from western regions (cfr. [156]). Archaeological finds from the Warring States period (475–221 BCE), including camel-decorated plaques and camel-shaped objects [111,125,157] (Figure 4a)—further indicate growing familiarity with these animals in Central China. Noteworthy, one gold and one silver camel of the Qin period (221–206 BCE), identical in form, were excavated from QLCM1 at the Mausoleum of the First Emperor Qin Shi Huang [158]. Osteological evidence documenting the presence of actual animals in this region is, however, scarce. This lack may partly relate to research bias in agriculturally focused regions such as the Central Plains, where faunal analyses—when conducted—have typically prioritized domesticates [40,41,159,160]. Nonetheless, currently available data identify only a single, poorly specified record, derived from the 1980s report on the bone workshop at Yuntang [70]. When considered against the numerous faunal investigations from the Central Plains (see [40,41] for a summary), this pattern suggests that interactions with camels in this region were likely largely indirect and possibly mediated through exchange networks rather than through sustained local use of live animals. Studies on the circulation of horses and other technologies during this period indicate that intermediary communities along the northern frontier facilitated exchanges between the Central Plains and the steppe and desert regions to the north, with the former showing variable rates of adoption or resistance [126,127]. A similar mediated dynamic may have shaped engagements with camels. However, unlike horses, which circulated more widely, the camel’s ecological and functional specialization likely constrained its broader expansion into the Central Plains.

Well-documented osteological evidence in Central China appears only in the Han period, most notably at the Pingling joint burial pit associated with Emperor Zhao of Han [83,84] and at the Xinmang coin-casting workshop in Nanyang [85]. This coincided with a marked increase in both the quantity and variety of camel imagery, including figurines, murals, textiles, and other media [22,110] (Figure 5) with representations ranging from some stylized or exaggerated forms [112,161] (Figure 4b) to notably realistic depictions [15,20,21] (Figure 4f). When considered alongside the archaeological presence of some camel remains, this realism suggests that at least some artisans could have become familiar with these animals through firsthand observation. Textual sources do indicate increasingly direct contacts during this period, with bamboo-slip documents from Xuanquan (Dunhuang, Gansu) recording camels entering the Han sphere after Zhang Qian’s western expeditions in the late first millennium BCE [108] and the *Hanshu* reporting that several Western polities presented Bactrian camels as tribute to the Han court. Despite a modest increase, osteological evidence from Central China remains notably limited and uneven compared to the north. While the scale of the Pingling joint burial pit deposit (33 individuals) indicates access to live camels and an organized logistical network, their concentration within a royal mortuary context more plausibly represents exceptional provisioning and/or gift circulation rather than routine economic use. If camels had been more fully integrated into everyday subsistence systems, clearer signals would be expected in residential or production faunal assemblages. Although zooarchaeological studies from both rural and urban residential sites in Han Central China are not especially numerous (but see [2,41,85,162,163]), only one site has so far reported camel remains [85]. As suggested by [128] this pattern is more consistent with these animals moving through relay-based exchange networks characteristic of much of the Han period. Their episodic appearance in the Central Plains likely resulted from targeted trade and gift exchange within complex political relationships with neighboring northern groups [164,165] rather than from broad incorporation into local daily systems.

During the medieval phase, the broader distribution of camel remains across northern China—including the northwest, the northeast, and, to a lesser extent, the Central Plains—was likely associated with an increasingly open political and economic context, which, particularly after the Tang dynasty, was characterized by more direct, intensified long-distance exchange in multiple directions [166,167], and, therefore, a growing demand for dependable pack animals. This shift is also evident in material culture, with a sharp increase in camel representations (Figure S1), most famously in Tang *sancai* figurines [e.g., [15,25,26], which portray them carrying people and goods including panniers, silk bundles, vessels, textiles, and felt rugs, and occasionally hunted game [15,20,128] (Figure 4c,d). This imagery suggests that by this time camels had likely become more broadly integrated into the Chinese socio-economic landscape, as communities in northern and central China remained closely engaged in trade, transport, and mobility [131,168,169]. During the Jin dynasty period (1115–1234 CE), the modern term for camel, *luotuo* (骆驼), was recorded by Zhang Hua in the *Bowu zhi* (博物志 Museum Records) [156] marking its formal establishment in scholarly and encyclopedic writing and, by extension, its incorporation into the cultural frameworks of all regions of China.

Nonetheless, the camel zooarchaeological record from the Central Plains remains scarce in comparison with northern regions. While research and recovery bias may partly contribute to this pattern, the limited presence of camel bones in residential faunal assemblages suggests that they were not widely maintained or used by local households and were unlikely to have formed part of routine economic life. Among the urban sites investigated in the Tang capital of Chang’an [62,91,170,171,172], only the West Market has yielded camel remains [91]. Even in later periods, camel bones occurring in Central Plains contexts often derive from urban commercial areas, whereas in northern regions they appear across a broader spectrum of archaeological contexts, including funerary and residential settings as well as state-linked mobility contexts such as markets and relay stations (Table 2). This distribution suggests that, while camels formed a more regular component of pastoral lifeways in the north, in the Central Plains they remained primarily associated with long-distance exchange and imperial logistical and political systems and functioned as “culturally familiar” transport animals of the arid frontier rather than part of everyday local practice. This perception, in many respects, persists into the present [13].

## 5. Conclusions

**Figure 7:**
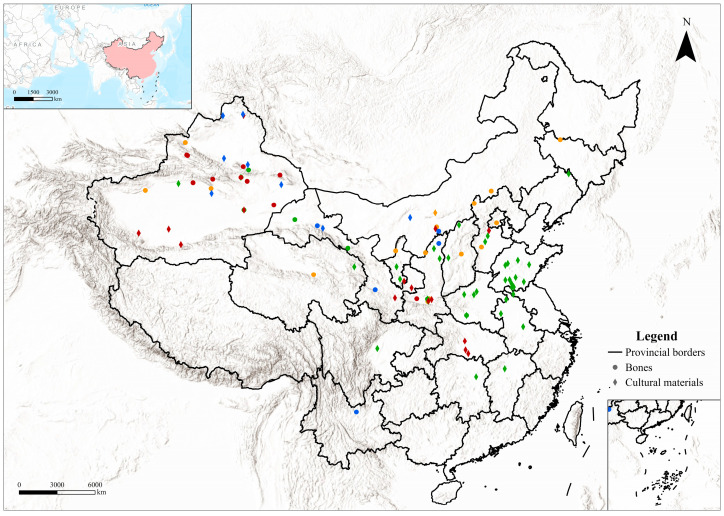
Distribution of cultural material representing camels and camel osteological evidence in different periods: Neolithic–Bronze Age (blue), Iron Age (red), Han period (green) and post-Han (yellow). Detailed information on the cultural material can be found in Table S1 and Figure S1. The map was created using ArcGIS Pro 3.0.2 (Esri; https://www.esri.com/en-us/arcgis/products/arcgis-pro/overview; last accessed on 19 January 2026). The basemap is the built-in Esri “World Topographic Map” (©2022 Esri Inc; 380 New York Street, Redlands, CA 92373, USA). Administrative boundaries were obtained from the Tianditu Cloud Center administrative division dataset (© Tianditu Cloud Center; managed by the National Geomatics Center of China under the Ministry of Natural Resources, Beijing, China; accessed on 13 January 2026; available at https://cloudcenter.tianditu.gov.cn/administrativeDivision).

Bactrian camels have been regarded in China as key drivers of long-distance connectivity [13,14,15]. By integrating osteoarchaeological data with broader archaeological and textual evidence, this study provides a more coherent framework for understanding human–camel interactions, showing that beyond their role in interregional exchange, camels also formed part of local economic systems in northern China, contributing to labor, subsistence, funerary practices, and craft production, albeit with regional and temporal variations (Figure 7, Figures S1 and S2).

Spatiotemporal patterns show that early camel remains and representations—primarily rock art—were concentrated in the arid and semi-arid zones of northwestern China, which correspond to the species’ preferred ecological settings [29,48]. From the first millennium BCE, camel imagery, mainly in the form of camel-decorated and camel-shaped objects, began to appear in central regions, likely resulting from intensified—though largely indirect—interaction with northern areas [20,21,22,23,173,174]. During the Han period, secure osteological evidence shows a gradual increase in Central China, alongside a wider range of textual references and more realistic representations in the material culture and texts, suggesting closer—though still sporadic—contact with live animals. Such encounters likely occurred through tributary exchanges and diplomatic gifting within broader political relations with northern neighbors [164,165]. These developments culminated in the expansion of Silk Road exchange networks, reaching their height in the Tang dynasty, when both a wider spread of camel skeletal remains and an exceptionally large and varied corpus of camel imagery [15,25,26] likely coincide with strongly increased long-distance exchange in multiple directions [166,167]. The same trend is observed in later periods, as Chinese communities remained closely engaged in trade, transport, and mobility [168,169]. Nevertheless, despite modest increases in Central China over time, camels remained primarily concentrated in the north (Figure 7 and Figure S2).

This pattern is best understood through a combination of environmental and economic considerations. Camels are optimally adapted to arid, desert, and semi-desert environments of Central Asia and far northern China, where they could be effectively raised and integrated into everyday pastoral practices [16,17,18,19,29,48]. Here they served as familiar working animals used for transport, subsistence, and other functions, accounting for the abundance of osteological remains and the relative scarcity of imagery—not due to lesser importance, but because they formed part of daily life rather than being exotic subjects.

While camels did reach the Central Plains, osteological evidence indicates that their presence was largely episodic, associated with targeted exchanges, and operating within imperial logistical and political systems rather than sustained local use. At the same time, their increased representations in material culture shows that their conceptual significance became more firmly incorporated into cultural, artistic, and socio-ideological repertoires. This pattern is not fully unexpected as the adoption of many new practices and technologies in the Central Plains was shaped less by the intrinsic properties of the innovations than by local suitability and social demand [126,175]. Camels—well adapted to arid environments but poorly suited to agricultural landscapes—were never fully incorporated into everyday life in Central China. Instead, they became closely associated with state transport systems, while acquiring broader cultural significance through their role in long-distance exchange networks.

Although this study advances our understanding of human–camel interactions in China, its results must be interpreted in light of persistent spatial and chronological research biases [40,43,62,63], as well as methodological difficulties in distinguishing camel species osteologically. The latter, in particular, affect not only interpretations of Bactrian camels but also assessments of possible dromedary presence in China, for which textual sources and cultural material provide limited yet intriguing evidence. The *Wei Shu* (魏书, Book of Wei) records that during the Zhengping reign (451–452 CE, present-day Shanxi) an envoy from a polity near Sogdia—generally identified with Mimi City (Penjikent) in present-day Uzbekistan—presented a “one-humped black camel” as tribute (cfr. [128]). Dromedaries also sporadically appear in Tang-period figurines [24,176,177,178] (Figure 4e). To date, however, no osteological remains from China have been identified as dromedaries, and the discrepancy between textual, visual, and skeletal evidence remains unresolved.

## Figures and Tables

**Table 1:** Mainstream historical chronology of northern China and chronological framework adopted in this study.

Dynasty/Kingdom	Age	Group
Yangshao	5000–3000 BCE	Neolithic–Bronze Age
Longshan	3000–1900 BCE	Neolithic–Bronze Age
Shang	1600–1046 BCE	Neolithic–Bronze Age
Western Zhou	1046–771 BCE	Iron Age
Eastern Zhou	771–256 BCE	Iron Age
Spring and Autumn	770–476 BCE	Iron Age
Warring States	475–221 BCE	Iron Age
Qin	221–206 BCE	Iron Age
Western Han	206 BCE–9 CE	Han
Xin	9–23 CE	Han
Eastern Han	25–220 CE	Han
Three kingdoms	220–280 CE	Post-Han
Jin	266–420 CE	Post-Han
Northern and Southern dynasties	420–589 CE	Post-Han
Sui	581–618 CE	Post-Han
Tang	618–907 CE	Post-Han
Five Dynasties and Ten Kingdoms	907–979 CE	Post Han
Yuan	1271–1368 CE	Post-Han
Ming	1368–1644 CE	Post-Han
Qing	1644–1912 CE	Post-Han

**Table 2:** Osteological evidence for camels in China (NB = Neolithic–Bronze Age; IA = Iron Age; H = Han period; PH = Post-Han period).

Site	Context	Location	Chronology	Stage	Camel Findings	Reference
Siwa 寺洼	Settlement	Upper Beiluo River, Shaanxi	Neolithic	NB	1 bone (*Camelus* sp.)	[38,63]
Wuding Xincun 武定新村	Cemetery	Wuding County, Chuxiong, Yunnan	Neolithic	NB	1 left astragalus (*Camelus* sp.)	[65]
Muzhuzhuliang 木柱柱梁	Settlement	Shenmu, Yulin, Shaanxi	Neolithic	NB	1 maxilla; 1 metatarsal (*Camelus* sp.)	[66]
Zhukaigou 朱开沟	Settlement and cemetery	Ejin Horo Banner, Ordos, Inner Mongolia	Neolithic–Bronze Age	NB	1 upper molar (*Camelus* sp.)	[67]
Huoshaogou 火烧沟	Cemetery	Yumen, Jiuquan, Gansu	Neolithic/Iron Age	NB/IA	Bones (Unspecified) (*Camelus* sp.)	[68,69]
Yuntang 云塘	Production site	Fufeng County, Baoji, Shaanxi	Iron Age	IA	Bones (Unspecified)	[70]
Xiaodonggou Nankou 小东沟南口	Cemetery	Hami, Xinjiang	Iron Age	IA	Bones (Unspecified)	[71]
Qunbake No. 2 群巴克二号	Cemetery	Luntai County, Bayingolin, Xinjiang	Iron Age	IA	Skulls	[72]
Xiaoxigou 小西沟	Settlement	Jimsar County, Changji, Xinjiang	Iron Age	IA	Bones (Unspecified)	[73]
Goubei 沟北	Cemetery	Turfan, Xinjiang	Iron Age	IA	Individuals	[74]
Jialekesikayinte 加勒克斯卡茵特	Cemetery	Nilka County, Ili, Xinjiang	Iron Age	IA	1 skull	[75]
Sangeqiao 三个桥	Cemetery	Dushan County, Ili, Xinjiang	Iron Age	IA	Bones (Unspecified)	[76]
Heishanling 黑山岭	Production site	Ruoqiang County, Bayingolin, Xinjiang	Iron Age	IA	ca. 10 bones	[61]
Shirenzigou 石人子沟	Settlement and cemetery	Barkol, Hami, Xinjiang	Iron Age	IA	1 individual and 5 single bones	[37]
Jirentai Goukou 吉仁台沟口	Cemetery	Nilka County, Ili, Xinjiang	Iron Age	IA	Bones (Unspecified)	[77]
Shichengzi 石城子	Military post	Qitai County, Changji, Xinjiang	Han	H	17 bones (including pelvis and lumbar vertebrae of *Camelus* sp.)	[78]
Sanjianfang 三间房	Administrative site	Ruoqiang County, Bayingolin, Xinjiang	Han	H	Bones (Unspecified)	[79]
Yuansha Ancient City 圆沙古城	Settlement	Yutian County, Hotan, Xinjiang	Han	H	531 bones	[80]
Xuanquanzhi 悬泉置	Post station	Dunhuang, Jiuquan, Gansu	Han	H	Bones (Unspecified)	[81]
Baguaying 八卦营	Cemetery	Baguaying, Zhangye City, Gansu	Han	H	Ribs and scapulas (Unspecified number)	[82]
Han Pingling Joint Burial Pit 汉平陵丛葬坑	Cemetery	Xianyang, Shaanxi	Han	H	33 individuals	[83,84]
Xinmang Coin-Casting Workshop 新莽铸钱作坊	Production site	Nanyang, Henan	Han	H	Bones and teeth (Unspecified)	[85]
Yuzigan Ancient City 玉孜干古城	Settlement	Korla, Bayingolin, Xinjiang	Wei–Jin	PH	Bones (Unspecified)	[86]
Tongwancheng West City 统万城西城	Settlement	Jingbian County, Yulin, Shaanxi	Eastern Jin	PH	1 maxilla, 2 mandibles, 1 humerus, 1 radius, 1 femur, 1 calcaneus, 1 carpal/tarsal	[87]
Yayidetimu 亚依德梯木	Beacon tower	Keping County, Aksu, Xinjiang	Tang	PH	Bones (Unspecified)	[88]
Weihe River Sand Pit Site 高陵渭河沙坑	Settlement	Gaoling District, Xi’an, Shaanxi	Tang	PH	1 damaged maxilla	[89]
Jinyang Ancient City晋阳古城	Settlement	Taiyuan, Shanxi	Tang	PH	1 phalanx	[90]
Chang’an West Market 长安西市	Production site (in the market)	Xi’an, Shaanxi	Tang	PH	5 bones	[91]
Harisai 哈日赛	Cemetery	Dulan County, Haixi, Qinghai	Tang	PH	4 individuals	[92]
Reshui 热水	Cemetery	Xuewei, Dulan County, Haixi, Qinghai	Tang	PH	Bones (Unspecified)	[93]
Kaiyuan Temple South Square 开元寺南广场	Settlement (Urban with market)	Zhengding County, Shijiazhuang, Hebei	Ming–Qing	PH	10 bones (Unspecified)	[38]
Dalete Ancient City 达勒特古城	Settlement	Bole, Bortala, Xinjiang	Song–Yuan	PH	Bones (Unspecified)	[94]
Daqingshan 大青山	Settlement (Urban)	Zhaoyuan County, Daqing, Heilongjiang	Liao–Jin	PH	3 bones	[95]
Minningcun 闽宁村	Funerary	Yongning County, Yinchuan, Ningxia	Western Xia	PH	1 individual	[24]
Guangyuanli 光源里	Ritual site	Xicheng District, Beijing	Jin	PH	2 bones	[38]
Xitucheng City 西土城城址	Settlement	Kangbao County, Zhangjiakou, Hebei	Jin	PH	31 bones	[96]
Yanjialiang 燕家梁	Settlement (Urban with market)	Baotou, Inner Mongolia	Yuan	PH	41 bones	[97]
Xiguanxiang 西关厢	Settlement (Commercial district)	Zhenglan, Xilingol League, Inner Mongolia	Yuan	PH	22 bones	[98]
Yilin Post Station 伊林驿站	Post station	Erenhot, Xilingol League, Inner Mongolia	Qing	PH	109 bones	[99]

## Data Availability

The original contributions presented in this study are included in the article. Further inquiries can be directed to the corresponding authors.

## Supplementary Materials

The following supporting information can be downloaded at: https://www.mdpi.com/article/10.3390/ani16050772/s1, Figure S1: Distribution of camel-related cultural materials in phase PH; Figure S2: Heat map showing the distribution of osteological and cultural evidence over time; Table S1: Details on material culture used in the maps.
