# Supplementary material for: Following Camels Between Bone and Culture: Camel–Human Interactions in China from the Neolithic to the Late Imperial Period
Source: Animals (Basel). 2026 Mar 1;16(5):772. doi: 10.3390/ani16050772 (PMC12984582; doi:10.3390/ani16050772)
Supplement: Supplementary file 1 [file animals-16-00772-s001.zip › animals-4158267-supplementary/animals-4158267-supplementary Captions Supplementary figures.pdf]

Figure S1. Distribution of camel-related cultural materials in phase PH. Detail data on the findings can be found in Table S1. The map was created using ArcGIS Pro 3.0.2 (Esri; https://www.esri.com/en-us/arcgis/products/arcgis-pro/overview). The basemap is the built-in Esri "World Topographic Map" (©2022 Esri Inc.). Administrative boundaries were obtained from the Tianditu Cloud Center administrative division dataset (© Tianditu Cloud Center; accessed on 13 January 2026; available at https://cloudcenter.tianditu.gov.cn/administrativeDivision).

Figure S2. Heat map showing the distribution of osteological and cultural evidence over time. Density index was generated in ArcGIS Pro using the Kernel Density tool (KDE) from point locations in WGS 1984 Web Mercator (Auxiliary Sphere) (cell size = 20,000 m; search radius = 300,000 m; population field = None, weight = 1). The output was masked to the China boundary and log10-transformed for visualization; zero/NoData areas are shown in white.
