# Supplementary material for: Following Camels Between Bone and Culture: Camel–Human Interactions in China from the Neolithic to the Late Imperial Period
Source: Animals (Basel). 2026 Mar 1;16(5):772. doi: 10.3390/ani16050772 (PMC12984582; doi:10.3390/ani16050772)
Supplement: Supplementary file 1 [file animals-16-00772-s001.zip › animals-4158267-supplementary/animals-4158267-supplementary Table_S1_New.pdf]

TableS1: Details on material culture used in the maps.

Site	Name in Chinese	Location	Age	Type of finding	Reference
Fangwanggang Tomb M1	安徽巢湖放王岗 M1	Chaohu, Anhui	Western Han	Gilt-bronze camel-shaped mat weight	[1]
Fangwanggang Tomb M1	安徽巢湖放王岗 M1	Chaohu, Anhui	Western Han	Gilt-bronze camel-shaped mat weight	[1]
Fangwanggang Tomb M1	安徽巢湖放王岗 M1	Chaohu, Anhui	Western Han	Gilt-bronze camel-shaped mat weight	[1]
Fangwanggang Tomb M1	安徽巢湖放王岗 M1	Chaohu, Anhui	Western Han	Gilt-bronze camel-shaped mat weight	[1]
Tomb of the Marquis of Ruyin	安徽阜阳双古堆西汉汝阴侯墓	Fuyang, Anhui	Western Han	Gilt-bronze plaque with camel motif	[2]
Jingzhou Ancient City	甘肃平凉泾川泾州古城	Jingchuan County, Pingliang, Gansu	Eastern Han	Bronze seal with camel-shaped knob	[3]

Liangyi Village	甘肃平凉良邑村	Jingchuan County, Pingliang, Gansu	Warring States	Gilt-bronze plaque with a "Hu people playing with a camel" motif	[4]
Tianshui Museum (collection)	甘肃天水博物馆藏	Tianshui, Gansu	Warring States	Silver camel-shaped applique ornament	[5]
Tianshui Museum (collection)	甘肃天水博物馆藏	Tianshui, Gansu	Warring States	Silver camel-shaped applique ornament	[5]
Tianshui Museum (collection)	甘肃天水博物馆藏	Tianshui, Gansu	Warring States	Silver camel-shaped applique ornament	[5]
Tianshui Museum (collection)	甘肃天水博物馆藏	Tianshui, Gansu	Warring States	Silver camel-shaped applique ornament	[5]
Pingling, Tomb of Emperor Zhao of Han: Outer Storage Pit No. 3	汉昭帝平陵三号外藏坑	Xianyang area, Shaanxi	Western Han	Wooden model of a chariot drawn by Bactrian camels	[6]

Pingling, Tomb of Emperor Zhao of Han: Outer Storage Pit No. 3	汉昭帝平陵三号外藏坑	Xianyang area, Shaanxi	Western Han	Wooden model of a chariot drawn by Bactrian camels	[6]
Pingling, Tomb of Emperor Zhao of Han: Outer Storage Pit No. 3	汉昭帝平陵三号外藏坑	Xianyang area, Shaanxi	Western Han	Wooden model of a chariot drawn by Bactrian camels	[6]
Pingling, Tomb of Emperor Zhao of Han: Outer Storage Pit No. 3	汉昭帝平陵三号外藏坑	Xianyang area, Shaanxi	Western Han	Wooden model of a chariot drawn by Bactrian camels	[6]
Mancheng Tomb M2	河北保定满城二号汉墓	Mancheng County, Baoding, Hebei	Western Han	Gold- and silver-inlaid bronze Boshan censer with camel motif	[7]
Sanpanshan Tomb M122	河北定县三盘山 M122	Dingzhou (Dingxian), Baoding, Hebei	Western Han	Gold- and silver-inlaid bronze chariot parasol-handle ring	[8]
Xinzhuangtou Tomb M30	河北易县辛庄头墓地 M30	Yi County, Baoding, Hebei	Warring States	Circular gold ornamental plaque with camel motif	[9]

Xindian Eastern Han tomb	河南洛阳辛店东汉墓	Luoyang, Henan	Eastern Han	Bronze seal with camel-shaped knob	[10]
Huangtushan Tomb M2	河南永城黄土山二号墓	Yongcheng, Henan	Western Han	Gold- and silver-inlaid bronze chariot parasol-handle ring	[11]
Wangshan Chu Tomb	湖北江陵望山楚墓	Jiangling County, Jingzhou, Hubei	Warring States	Camel-rider-shaped bronze lamp	[12]
Hougang Chu Tomb	湖北荆门后港楚墓	Jingmen, Hubei	Warring States	Camel-rider-shaped bronze lamp	[13]
Yangjiashan Tomb M30,	湖南长沙杨家山 M304	Changsha, Hunan	Western Han	Gold-foil applique with camel motif	[14]
Tomb of Liu He	江西南昌海昏侯刘贺墓	Nanchang, Jiangxi	Western Han	Gilt camel-shaped hook	[15]
Tomb of Liu He	江西南昌海昏侯刘贺墓	Nanchang, Jiangxi	Western Han	Gilt camel-shaped hook	[15]
Tomb of Liu He	江西南昌海昏侯刘贺墓	Nanchang, Jiangxi	Western Han	Gilt camel-shaped hook	[15]

Tomb of Liu He	江西南昌海昏侯刘贺墓	Nanchang, Jiangxi	Western Han	Gilt camel-shaped hook	[15]
Jingzhou Museum (collection)	荆州博物馆藏	Jingzhou, Hubei	Warring States	Camel-shaped bronze lamp	[16]
Xichagou	辽宁西丰西岔沟	Xifeng County, Tieling, Liaoning	Western Han	Bronze ornamental plaque	[17]
Ordos region, Inner Mongolia (unspecified)	内蒙古鄂尔多斯地区	Ordos, Inner Mongolia	Warring States	Bronze mirror with handle decorated with a rider-on-camel motif	[18]
Ordos region, Inner Mongolia (unspecified)	内蒙古鄂尔多斯地区	Ordos, Inner Mongolia	Spring and Autumn–Warring States	Bronze mirror with camel-shaped handle	[19]
Ordos region, Inner Mongolia (unspecified)	内蒙古鄂尔多斯地区	Ordos, Inner Mongolia	Western Han	Camel-shaped bronze ornament	[20]

Ordos region, Inner Mongolia (collection; provenance unknown)	内蒙古鄂尔多斯地区（馆藏/出土信息不详）	Ordos, Inner Mongolia	Spring and Autumn–Eastern Han (broad)	Gilt-bronze ornamental plaque with a "Hu people playing with a camel" motif	[21]
Ordos region, Inner Mongolia (collection; provenance unknown)	内蒙古鄂尔多斯地区（馆藏/出土信息不详）	Ordos, Inner Mongolia	Spring and Autumn–Eastern Han (broad)	Gilt-bronze ornamental plaque with a "Hu people playing with a camel" motif	[21]
Ordos region, Inner Mongolia (collection; provenance unknown)	内蒙古鄂尔多斯地区（馆藏/出土信息不详）	Ordos, Inner Mongolia	Spring and Autumn–Eastern Han (broad)	Gilt-bronze ornamental plaque with a "Hu people playing with a camel" motif	[21]
Ordos region, Inner Mongolia (collection; provenance unknown)	内蒙古鄂尔多斯地区（馆藏/出土信息不详）	Ordos, Inner Mongolia	Spring and Autumn–Eastern Han (broad)	Gilt-bronze ornamental plaque with a "Hu people playing with a camel" motif	[21]
Ordos region, Inner Mongolia (collection; provenance unknown)	内蒙古鄂尔多斯地区（馆藏/出土信息不详）	Ordos, Inner Mongolia	Spring and Autumn–Eastern Han (broad)	Gilt-bronze ornamental plaque with human–tiger–camel motif	[21]

Ordos region, Inner Mongolia (collection; provenance unknown)	内蒙古鄂尔多斯地区（馆藏/出土信息不详）	Ordos, Inner Mongolia	Spring and Autumn–Eastern Han (broad)	Gilded bronze ornamental plaque with twin-camel motif	[21]
Ordos region, Inner Mongolia (collection; provenance unknown)	内蒙古鄂尔多斯地区（馆藏/出土信息不详）	Ordos, Inner Mongolia	Spring and Autumn–Eastern Han (broad)	Bronze ornamental plaque with twin-camel motif	[21]
Ordos region, Inner Mongolia (collection; provenance unknown)	内蒙古鄂尔多斯地区（馆藏/出土信息不详）	Ordos, Inner Mongolia	Spring and Autumn–Eastern Han (broad)	Bronze ornamental plaque with camel motif	[21]
Ordos region, Inner Mongolia (collection; provenance unknown)	内蒙古鄂尔多斯地区（馆藏/出土信息不详）	Ordos, Inner Mongolia	Spring and Autumn–Eastern Han (broad)	Bronze ornamental plaque with camel motif (one of a pair)	[21]
Ordos region, Inner Mongolia (collection; provenance unknown)	内蒙古鄂尔多斯地区（馆藏/出土信息不详）	Ordos, Inner Mongolia	Spring and Autumn–Eastern Han (broad)	Bronze ornamental plaque with camel motif (one of a pair)	[21]

Ordos region, Inner Mongolia (collection; provenance unknown)	内蒙古鄂尔多斯地区（馆藏/出土信息不详）	Ordos, Inner Mongolia	Spring and Autumn–Eastern Han (broad)	Bronze plaque with twin-camel motif	[21]
Yimeng League (Ikh Juu League) region, Inner Mongolia (Unspecified)	内蒙古伊盟（伊克昭盟）	Ordos area, Inner Mongolia	Han dynasty	Bronze seal with camel-shaped knob	[22]
Tomb M3, Western Han Tomb Group III (suburban area)	宁夏固原城郊西汉墓IIIM3	Guyuan, Ningxia	Western Han	Bronze camel-shaped mat weight	[23]
Tomb M3, Western Han Tomb Group III (suburban area)	宁夏固原城郊西汉墓IIIM3	Guyuan, Ningxia	Western Han	Bronze camel-shaped mat weight	[23]
Tomb M3, Western Han Tomb Group III (suburban area)	宁夏固原城郊西汉墓IIIM3	Guyuan, Ningxia	Western Han	Bronze camel-shaped mat weight	[23]
Zhangjiecun Xirong Cemetery	宁夏彭阳张街村春秋战国西戎墓地（采集）	Pengyang County, Guyuan, Ningxia	Spring and Autumn–Warring States	Copper-alloy ornamental plaque with human-and-camel motif	[24]

Daodunzi Xiongnu Cemetery	宁夏同心倒墩子西汉匈奴墓地	Tongxin County, Wuzhong, Ningxia	Western Han	Copper-alloy ornamental plaque with twin-camel motif	[25]
Daodunzi Xiongnu Cemetery	宁夏同心倒墩子西汉匈奴墓地	Tongxin County, Wuzhong, Ningxia	Western Han	Copper-alloy ornamental plaque with twin-camel motif	[25]
Mausoleum of the First Emperor Qin Shi Huang QLCM1	秦始皇帝陵 QLCM1	Lintong District, Xi'an, Shaanxi	Qin dynasty	Gold camel-shaped figurine	[26]
Mausoleum of the First Emperor Qin Shi Huang QLCM1	秦始皇帝陵 QLCM1	Lintong District, Xi'an, Shaanxi	Qin dynasty	Silver camel-shaped figurine	[26]
Western Cemetery, Tomb M1 (QLCM1), Mausoleum of the First Emperor Qin Shi Huang	秦始皇帝陵陵西墓葬 M1（QLCM1）	Lintong District, Xi'an, Shaanxi	Qin dynasty	Gold ornamental plaque with camels motif	[27]
Shangsunjiatai Cemetery Area Yi Tomb M1,	青海大通上孙家寨乙 M1	Datong Hui and Tu Autonomous County, Xining, Qinghai	Eastern Han	Bronze seal with camel-shaped knob	[27]

Anqiu, Shandong (stray find)	山东安丘	Anqiu, Weifang, Shandong	Han dynasty	Bronze camel-shaped mat weight	[28]
Tomb of the King of Qi	山东淄博西汉齐王墓	Zibo, Shandong	Western Han	Gilt-bronze hemispherical *jieyue* (horse harness) fitting with camel motif	[29]
Tomb of the King of Qi	山东淄博西汉齐王墓	Zibo, Shandong	Western Han	Gilt-bronze hemispherical *jieyue* (horse harness) fitting with camel motif	[29]
Tomb of the King of Qi	山东淄博西汉齐王墓	Zibo, Shandong	Western Han	Gilt-bronze hemispherical *jieyue* (horse harness) fitting with camel motif	[29]
Tomb of the King of Qi	山东淄博西汉齐王墓	Zibo, Shandong	Western Han	Gilt-bronze hemispherical *jieyue* (horse harness) fitting with camel motif	[29]
Tomb of the King of Qi	山东淄博西汉齐王墓	Zibo, Shandong	Western Han	Gilt-bronze hemispherical *jieyue* (horse harness) fitting with camel motif	[29]
Tomb of the King of Qi	山东淄博西汉齐王墓	Zibo, Shandong	Western Han	Gilt-bronze hemispherical *jieyue* (horse harness) fitting with camel motif	[29]

Tomb of the King of Qi	山东淄博西汉齐王墓	Zibo, Shandong	Western Han	Gilt-bronze hemispherical *jieyue* (horse harness) fitting with camel motif	[29]
Tomb of the King of Qi	山东淄博西汉齐王墓	Zibo, Shandong	Western Han	Gilt-bronze hemispherical *jieyue* (horse harness) fitting with camel motif	[29]
Tomb of the King of Qi	山东淄博西汉齐王墓	Zibo, Shandong	Western Han	Gilt-bronze hemispherical *jieyue* (horse harness) fitting with camel motif	[29]
Tomb of the King of Qi	山东淄博西汉齐王墓	Zibo, Shandong	Western Han	Gilt-bronze hemispherical *jieyue* (horse harness) fitting with camel motif	[29]
Tomb of the King of Qi	山东淄博西汉齐王墓	Zibo, Shandong	Western Han	Gilt-bronze hemispherical *jieyue* (horse harness) fitting with camel motif	[29]
Tomb of the King of Qi	山东淄博西汉齐王墓	Zibo, Shandong	Western Han	Gilt-bronze hemispherical *jieyue* (horse harness) fitting with camel motif	[29]
Tomb of the King of Qi	山东淄博西汉齐王墓	Zibo, Shandong	Western Han	Gilt-bronze hemispherical *jieyue* (horse harness) fitting with camel motif	[29]

Tomb of the King of Qi	山东淄博西汉齐王墓	Zibo, Shandong	Western Han	Gilt-bronze hemispherical *jieyue* (horse harness) fitting with camel motif	[29]
Tomb of the King of Qi	山东淄博西汉齐王墓	Zibo, Shandong	Western Han	Gilt-bronze hemispherical *jieyue* (horse harness) fitting with camel motif	[29]
Tomb of the King of Qi	山东淄博西汉齐王墓	Zibo, Shandong	Western Han	Gilt-bronze hemispherical *jieyue* (horse harness) fitting with camel motif	[29]
Tomb of the King of Qi	山东淄博西汉齐王墓	Zibo, Shandong	Western Han	Gilt-bronze hemispherical *jieyue* (horse harness) fitting with camel motif	[29]
Tomb of the King of Qi	山东淄博西汉齐王墓	Zibo, Shandong	Western Han	Gilt-bronze hemispherical *jieyue* (horse harness) fitting with camel motif	[29]
Tomb of the King of Qi	山东淄博西汉齐王墓	Zibo, Shandong	Western Han	Gilt-bronze hemispherical *jieyue* (horse harness) fitting with camel motif	[29]
Tomb of the King of Qi	山东淄博西汉齐王墓	Zibo, Shandong	Western Han	Gilt-bronze hemispherical *jieyue* (horse harness) fitting with camel motif	[29]

Tomb of the King of Qi	山东淄博西汉齐王墓	Zibo, Shandong	Western Han	Gilt-bronze hemispherical *jieyue* (horse harness) fitting with camel motif	[29]
Tomb of the King of Qi	山东淄博西汉齐王墓	Zibo, Shandong	Western Han	Gilt-bronze hemispherical *jieyue* (horse harness) fitting with camel motif	[29]
Tomb of the King of Qi	山东淄博西汉齐王墓	Zibo, Shandong	Western Han	Gilt-bronze hemispherical *jieyue* (horse harness) fitting with camel motif	[29]
Tomb of the King of Qi	山东淄博西汉齐王墓	Zibo, Shandong	Western Han	Gilt-bronze hemispherical *jieyue* (horse harness) fitting with camel motif	[29]
Tomb of the King of Qi	山东淄博西汉齐王墓	Zibo, Shandong	Western Han	Gilt-bronze hemispherical *jieyue* (horse harness) fitting with camel motif	[29]
Tomb of the King of Qi	山东淄博西汉齐王墓	Zibo, Shandong	Western Han	Gilt-bronze hemispherical *jieyue* (horse harness) fitting with camel motif	[29]
Tomb of the King of Qi	山东淄博西汉齐王墓	Zibo, Shandong	Western Han	Gilt-bronze hemispherical *jieyue* (horse harness) fitting with camel motif	[29]

Tomb of the King of Qi	山东淄博西汉齐王墓	Zibo, Shandong	Western Han	Gilt-bronze hemispherical *jieyue* (horse harness) fitting with camel motif	[29]
Tomb of the King of Qi	山东淄博西汉齐王墓	Zibo, Shandong	Western Han	Gilt-bronze hemispherical *jieyue* (horse harness) fitting with camel motif	[29]
Tomb of the King of Qi	山东淄博西汉齐王墓	Zibo, Shandong	Western Han	Gilt-bronze hemispherical *jieyue* (horse harness) fitting with camel motif	[29]
Tomb of the King of Qi	山东淄博西汉齐王墓	Zibo, Shandong	Western Han	Gilt-bronze hemispherical *jieyue* (horse harness) fitting with camel motif	[29]
Tomb of the King of Qi	山东淄博西汉齐王墓	Zibo, Shandong	Western Han	Gilt-bronze hemispherical *jieyue* (horse harness) fitting with camel motif	[29]
Tomb of the King of Qi	山东淄博西汉齐王墓	Zibo, Shandong	Western Han	Gilt-bronze hemispherical *jieyue* (horse harness) fitting with camel motif	[29]
Tomb of the King of Qi	山东淄博西汉齐王墓	Zibo, Shandong	Western Han	Gilt-bronze hemispherical *jieyue* (horse harness) fitting with camel motif	[29]

Tomb of the King of Qi	山东淄博西汉齐王墓	Zibo, Shandong	Western Han	Gilt-bronze hemispherical *jieyue* (horse harness) fitting with camel motif	[29]
Tomb of the King of Qi	山东淄博西汉齐王墓	Zibo, Shandong	Western Han	Gilt-bronze hemispherical *jieyue* (horse harness) fitting with camel motif	[29]
Tomb of the King of Qi	山东淄博西汉齐王墓	Zibo, Shandong	Western Han	Gilt-bronze hemispherical *jieyue* (horse harness) fitting with camel motif	[29]
Tomb of the King of Qi	山东淄博西汉齐王墓	Zibo, Shandong	Western Han	Gilt-bronze hemispherical *jieyue* (horse harness) fitting with camel motif	[29]
Tomb of the King of Qi	山东淄博西汉齐王墓	Zibo, Shandong	Western Han	Gilt-bronze hemispherical *jieyue* (horse harness) fitting with camel motif	[29]
Tomb of the King of Qi	山东淄博西汉齐王墓	Zibo, Shandong	Western Han	Gilt-bronze hemispherical *jieyue* (horse harness) fitting with camel motif	[29]
Tomb of the King of Qi	山东淄博西汉齐王墓	Zibo, Shandong	Western Han	Gilt-bronze hemispherical *jieyue* (horse harness) fitting with camel motif	[29]

Tomb of the King of Qi	山东淄博西汉齐王墓	Zibo, Shandong	Western Han	Gilt-bronze hemispherical *jieyue* (horse harness) fitting with camel motif	[29]
Tomb of the King of Qi	山东淄博西汉齐王墓	Zibo, Shandong	Western Han	Gilt-bronze hemispherical *jieyue* (horse harness) fitting with camel motif	[29]
Dachuan Village	山西右玉县大川村	Youyu County, Shuozhou, Shanxi	Western Han	Wine container *zun* with camel motif	[29]
Dachuan Village	山西右玉县大川村	Youyu County, Shuozhou, Shanxi	Western Han	Wine container *zun* with camel motif	[30]
Dachuan Village	山西右玉县大川村	Youyu County, Shuozhou, Shanxi	Western Han	Wine container *zun* with camel motif	[30]
Northern Shaanxi (Unspecified)	陕西陕北（具体地点不详）	Shaanxi	Western Han	Gilt-bronze ornamental plaque with twin-camel motif (one of a pair)	[30]

Northern Shaanxi (Unspecified)	陕西陕北（具体地点不详）	Shaanxi	Western Han	Gilt-bronze ornamental plaque with twin-camel motif (one of a pair)	[21:a]
Tomb M1, Gobeimudi Cemetery, Jiaohe Ancient City	吐鲁番交河故城沟北墓地 M1	Turfan, Xinjiang	Western Han	Camel-shaped gold applique	[21:a]
Tomb M1, Gobeimudi Cemetery, Jiaohe Ancient City	吐鲁番交河故城沟北墓地 M1	Turfan, Xinjiang	Western Han	Camel-shaped gold applique	[31]
Longshoucun	西安北郊龙首村	Xi'an, Shaanxi	Warring States–Western Han	Gold plaque with twin-camel motif	[31]
Youjiazhuang Han Tomb	西安北郊尤家庄汉墓	Xi'an, Shaanxi	Han dynasty	Glazed pottery camel figurine	[32]
Shapocun Brickyard Western Han Tomb	西安沙坡村砖瓦厂西汉墓	Xi'an, Shaanxi	Western Han	Painted pottery camel figurine	[33]

Shapocun Brickyard Western Han tomb	西安沙坡村砖瓦厂西汉墓	Xi'an, Shaanxi	Western Han	Painted pottery camel figurine	[33]
Tarlang	新疆阿勒泰塔尔浪	Altay, Xinjiang	Spring and Autumn–Western Han	Bronze camel-shaped knife handle	[33]
Niya	新疆和田尼雅遗址	Minfeng County, Hotan Xinjiang	Han–Jin	Textile fragment decorated with camel-motif	[34]
Niya	新疆和田尼雅遗址	Minfeng County, Hotan Xinjiang	Han–Jin	Textile fragment decorated with camel-motif	[35]

Shanpula Cemetery	新疆和田山普拉墓地	Hotan, Xinjiang	Han–Jin	Textile fragment decorated with camel-motif	[35]
Shanpula Cemetery	新疆和田山普拉墓地	Hotan, Xinjiang	Han–Jin	Textile fragment decorated with camel-motif	[35]
Yotkan	新疆和田约特干遗址	Hotan City, Xinjiang	Han dynasty	Copper-alloy ornamental plaque with camel motif	[35]

Zagunluk Cemetery No. 1 Tomb 96QZIM17	新疆且末扎滚鲁克一号墓地 96QZIM17	Qiemo County, Bayingolin, Xinjiang	Spring and Autumn–Warring States	Wooden bucket incised with a twin-camels motif	[36]
West bank of Lop Nor (surface collection)	新疆若羌罗布泊西岸（采集）	Ruoqiang County, Bayingolin, Xinjiang	Warring States	Copper-alloy ornamental plaque with camel motif	[37]
Yuqikate	新疆新和县玉奇喀特乡	Xinhe County, Aksu, Xinjiang	Han dynasty	Bronze seal with camel-shaped knob	[38]
Tomb of Sima Jinlong	山西大同石家寨北魏司马金龙墓	Pingcheng District, Datong, Shanxi	Northern Wei	Figurine of a Hu man leading a camel	[39]
Chenzhuang Northern Wei Tomb	山西大同县陈庄北魏墓	Yunzhou District, Datong, Shanxi	Northern Wei	Fragmentary figurine of a Hu man leading a camel	[40]
Wenying Road Northern Wei mural tomb	山西大同文瀛路北魏壁画墓	Pingcheng District, Datong, Shanxi	Northern Wei	Mural of a Hu man leading a camel	[41]
Yanshi Northern Wei Tomb	河南洛阳偃师北魏墓	Yanshi District, Luoyang, Henan	Northern Wei	Pottery camel figurine	[42]
Sha Factory West Road HM555	河南洛阳纱厂西路	Xigong District,	Northern Wei	Pottery camel figurine	[43]

	HM555	Luoyang, Henan			
Hengshan Road Northern Wei Tomb	河南洛阳涧西衡山路北魏墓	Jianxi District, Luoyang, Henan	Northern Wei	Pottery camel figurine	[44]
Jili District Northern Wei Tomb	河南洛阳吉利区北魏墓	Ji Li District, Luoyang, Henan	Northern Wei	Pottery camel figurine	[45]
Shenggou Village Northern Zhou Li Xian Tomb	宁夏固原南郊乡深沟村北周李贤墓	Yuanzhou District, Guyuan, Ningxia	Northern Zhou	Pottery camel figurine	[46]
Zhongbao Village Tang Tomb	陕西西安西郊中堡村唐墓	Lianhu District, Xi'an, Shaanxi	Tang	*Sancai* camel figurine carrying musicians	[47]
Xi'an southern suburbs Tomb M31	陕西西安南郊 M31 唐墓	Yanta District, Xi'an, Shaanxi	Tang	*Sancai*-glazed camel figurine with a beast-head pannier ornament	[48]
Xi'an southern suburbs Tomb M31	陕西西安南郊 M31 唐墓	Yanta District, Xi'an, Shaanxi	Tang	Camel-riding musician figurine	[49]
Thermal Power Plant Tomb M31	陕西西安西郊热电厂唐墓 M31	Lianhu District, Xi'an, Shaanxi	Tang	*Sancai*-glazed camel figurine	[50]
Shaanxi Xi'an eastern suburbs Tomb M609	陕西西安东郊唐墓 M609	Baqiao District, Xi'an, Shaanxi	Tang	*Sancai*-glazed camel figurine	[51]

Joint Tomb of Han Xiu and his wife	陕西西安韩休夫妇墓	Chang'an District, Xi'an, Shaanxi	Tang	Pottery camel figurine	[52]
Maopo Village M21	陕西西安茅坡村 M21	Yanta District, Xi'an, Shaanxi	Late Sui to early Tang	Camel figurine with a pannier	[53]
Beizhu Village M11	陕西咸阳北杜镇北朱村 M11	Weicheng District, Xianyang, Shaanxi	Sui	Fragment of a camel figurine with pannier	[54]
Joint Tomb of Zhang Chen and his wife	陕西西安隋张綝夫妇合葬墓	Yanta District, Xi'an, Shaanxi	Sui, Daye 3 (607 CE)	Camel figurine	[55]
Tomb of Xianyu Tinghui	陕西西安开元十一年鲜于庭诲墓	Yanta District, Xi'an, Shaanxi	Tang	*Sancai*-glazed camel figurine carrying musicians	[56]
Tomb of Zheng Rentai	陕西礼泉昭陵郑仁泰墓	Liquan County, Xianyang, Shaanxi	Tang	Painted pottery camel figurine	[57]
Tomb of Prince Yi De	陕西乾县懿德太子墓	Qianxian, Xianyang, Shaanxi	Tang	*Sancai*-glazed camel figurine	[58]
Tomb of Prince Zhang Huai	陕西乾县章怀太子墓	Qianxian, Xianyang, Shaanxi	Tang	Pottery camel figurine	[59]
Tomb of Li Feng	陕西富平李凤墓	Fuping County,	Tang	Pottery camel figurine	[60]

		Weinan, Shaanxi			
Nanliwang Village Tomb	陕西长安县南里王村唐墓	Chang'an District, Xi'an, Shaanxi	Tang	Pottery camel figurine	[61]
Tomb of Sui Hu Lvche	山西太原隋斛律彻墓	Jinyuan District, Taiyuan, Shanxi	Sui	Camel figurine carrying silk, with a Hu rider seated on the camel	[62]
Tomb of Sui Yu Hong	山西太原隋虞弘墓	Jinyuan District, Taiyuan, Shanxi	Sui	Stone coffin relief of a Hu rider hunting on camelback	[63]
Zhijiabao Tang Tomb	山西大同智家堡唐墓	Pingcheng District, Datong, Shanxi	Tang	Pottery camel figurine	[64]
Tomb of Wang Shen Tomb	山西长治唐王琛墓	Changzhi, Shanxi	Tang	Pottery figurine of a Hu merchant riding a camel	[65]
North Street Tang Tomb	山西黎城县北街唐墓	Li Cheng County, Changzhi, Shanxi	Tang	Figurine of a Hu woman riding a camel	[65]
Hongxing Factory Tomb	山西长治市红星厂唐墓	Changzhi, Shanxi	Tang	Painted pottery figurine of a Hu figure holding a monkey	[65]
Changzhi Tang Tomb	山西长治唐墓	Lu Zhou District, Changzhi, Shanxi	Tang	Pottery camel figurine	[66]
Tomb of Guo Xiang	河北南和唐墓（郭	Nanhe District,	Tang	Pottery camel figurine	[67]

	祥墓）	Xingtai, Hebei			
Joint tomb of An Pu and his wife	河南洛阳龙门唐安菩夫妇墓	Luolong District, Luoyang, Henan	Tang, Jinglong 3 (709 CE)	*Sancai*-glazed camel figurine	[68]
Joint tomb of An Pu and his wife	河南洛阳龙门唐安菩夫妇墓	Luolong District, Luoyang, Henan	Tang, Jinglong 3 (709 CE)	*Sancai*-glazed figurine of a camel leader	[68]
Joint tomb of An Pu and his wife	河南洛阳龙门唐安菩夫妇墓	Luolong District, Luoyang, Henan	Tang, Jinglong 3 (709 CE)	Yellow-glazed camel figurine	[68]
Tomb of Princess Ruru	河北磁县东魏茹茹公主墓	Cixian, Handan, Hebei	Eastern Wei	Pottery camel figurine	[69]
Wanzhang Bei Chao mural tomb	河北磁县湾漳北朝壁画墓	Cixian, Handan, Hebei	Northern Dynasties	Pottery camel figurine	[70]
Tomb of Shi Siming	北京丰台唐史思明墓	Feng Tai District, Beijing	Tang	Pottery camel figurine	[71]
Chaoyang Tang Tomb	辽宁朝阳唐墓	Shuangta District, Chaoyang, Liaoning	Tang	Pottery camel figurine	[72]
Hohhot University East Road Northern Wei Tomb	内蒙古呼和浩特大学东路北魏墓	Saihan District, Hohhot, Inner	Northern Wei	Pottery camel figurine	[73]

		Mongolia			
Bayannur Tang Tomb	内蒙古巴彦诺尔唐墓	Bayannur, Inner Mongolia	Tang	Pottery camel figurine	[74]
Nanyuan Tomb M1	宁夏固原南塬唐墓 M1	Yuanzhou District, Guyuan, Ningxia	Tang	Pottery camel figurine	[75]
Nanyuan Tomb M3	宁夏固原南塬唐墓 M3	Yuanzhou District, Guyuan, Ningxia	Tang	Pottery camel figurine	[76]
Wang Lao Ba Tang Tomb	宁夏固原王涝坝唐墓	Yuanzhou District, Guyuan, Ningxia	Tang	Pottery camel figurine	[77]
Wuzhong western suburbs Tomb M4	宁夏吴忠西郊唐墓 M4	Litong District, Wuzhong, Ningxia	Tang	Pottery camel figurine	[78]
Yanchi Tomb M3	宁夏盐池唐墓 M3	Yanchi County, Wuzhong, Ningxia	Tang	Pottery camel figurine	[79]
Lianhu Farm Yuquanying Tang Tomb	宁夏青铜峡市莲湖农场玉泉营唐墓	Qingtongxia, Ningxia	Tang	Painted pottery camel figurine	[80]
Wuwei western suburbs Western Xia Tomb	甘肃武威西郊西夏墓	Liang Zhou District, Wuwei, Gansu	Western Xia	Wooden camel figurine	[81]

Zhangyi Western Xia Tomb	甘肃武威张义西夏墓	Tian Zhu County, Wuwei, Gansu	Western Xia	Camel painted on wooden panel	[82]
Tomb M11 of the Wang Shixian family	甘肃漳县徐家坪汪世显家族墓 M11	Zhangxian, Ding Xi, Gansu	Yuan	Pottery camel figurine	[83]
Tomb M14 of the Wang Shixian family	甘肃漳县徐家坪汪世显家族墓 M14	Zhangxian, Ding Xi, Gansu	Yuan	Wooden camel	[83]
Tomb M20 of the Wang Shixian family	甘肃漳县徐家坪汪世显家族墓 M20	Zhangxian, Ding Xi, Gansu	Yuan	Pottery camel figurine	[83]
Yinchuan Western Xia Mausoleum M177	宁夏银川西夏陵 M177	Xi Xia District, Yinchuan, Ningxia	Western Xia	Gilt-bronze camel	[84]
Ejin Horo Banner Western Xia Tomb	内蒙古伊金霍洛旗西夏墓	Ejin Horo Banner, Ordos, Inner Mongolia	Western Xia	Pottery camel figurine	[85]
Sanyanjing Yuan Tomb	内蒙古赤峰三眼井元墓	Yuanbaoshan District, Chifeng, Inner Mongolia	Yuan	Pottery camel figurine	[86]
Sanyanjing Yuan Tomb	内蒙古赤峰沙子山	Yuanbaoshan	Yuan	Pottery camel figurine	[87]

	元墓	District, Chifeng, Inner Mongolia			
Houyingfang	北京元大都后英房遗址	Xicheng District, Beijing	Yuan	Pottery camel figurine	[88]
Xitiao Hutong	北京元大都西绦胡同遗址	Xicheng District, Beijing	Yuan	Pottery camel figurine	[88]
Tomb of Wang Shiying	西安南郊元代王世英墓	Yanta District, Xi'an, Shaanxi	Yuan	Gray pottery camel figurine	[89]
Tomb of Liu Yishi	西安东郊元刘义世墓	Baqiao District, Xi'an, Shaanxi	Yuan	Pottery camel figurine	[90]
Tomb of Feng Daozhen	山西大同宋家庄冯道真墓	Pingcheng District, Datong, Shanxi	Yuan	Pottery camel figurine	[91]
Beiyukou Yuan Tomb	山西文水北峪口元墓	Wenshui County, Lv Liang, Shanxi	Yuan	Pottery camel figurine	[92]
Qujiang Yuan Tomb M1	西安曲江元墓 M1	Yanta District, Xi'an, Shaanxi	Yuan	Pottery camel figurine	[93]
Xiaoling Mausoleum	江苏南京明孝陵陪	Xuanwu District,	Ming	Pottery camel figurine	[94]

	葬墓	Nanjing, Jiangsu			
Tomb of Mu Ying	江苏南京沐英墓	Jiangning District, Nanjing, Jiangsu	Ming	Pottery camel figurine	[95]
Tomb of the Xuda family	江苏南京徐达家族墓	Xuanwu District, Nanjing, Jiangsu	Ming	Pottery camel figurine	[96]
Tomb of Dengyu	江苏南京邓愈墓	Yuhuatai District, Nanjing, Jiangsu	Ming	Pottery camel figurine	[97]
Dingling Mausoleum	北京昌平明定陵	Changping District, Beijing	Ming	Wooden camel figurine	[98]
Tomb of King Lu Huang	山东邹城鲁荒王墓	Zoucheng, Jining, Shandong	Ming	Wooden carved camel	[99]
Mausoleum of King Shu	四川成都明蜀王陵	Longquanyi District, Chengdu, Sichuan	Ming	Pottery camel figurine	[100]
Tomb of King Liang Zhuang	湖北钟祥明梁庄王墓	Zhongxiang, Hubei	Ming	Camel ornament on gold and silver ware	[101]
Tomb of King Ning Wang	江西南昌明宁王墓	Xinjian District, Nanchang, Jiangxi	Ming	Pottery camel figurine	[102]

The Palace Museum (collection)	北京故宫博物院	Dongcheng District, Beijing	Qing	White jade camel brush rest	[103]
The Palace Museum (collection)	北京故宫博物院	Dongcheng District, Beijing	Qing	Reclining camel carved in green jade	[104]
The Palace Museum (collection)	北京故宫博物院	Dongcheng District, Beijing	Qing	Ivory camel carved in ivory	[105]
National Palace Museum (collection)	台北故宫博物院	Shi Lin District, Taipei	Qing	Jade carving of a man herding a camel	[106]
Mountain Resort	河北承德避暑山庄	Shuangqiao District, Chengde, Hebei	Qing	Gilt-bronze camel	[107]
Puning Temple	河北承德普宁寺	Shuangqiao District, Chengde, Hebei	Qing	Wooden carved camel	[108]
Norbulingka (collection)	西藏拉萨罗布林卡	Chengguan District, Lhasa, Tibet	Qing	Gilt-bronze camel	[109]
Inner Mongolia Ordos Otog Banner (collection)	内蒙古鄂尔多斯鄂托克旗	Otog Banner, Ordos, Inner Mongolia	Qing	Silver camel ornament	[110]
Inner Mongolia Xilin Gol	内蒙古锡林郭勒盟	Xilin Gol League,	Qing	Camel on felt	[111]

League (collection)		Inner Mongolia			
Kashgar Prefecture (collection)	新疆喀什地区民间征集	Kashgar Prefecture, Xinjiang	Qing	Camel-hair woven carpet	[112]
Lady Tang, consort of Prince Xiang Li Dan of Anguo	河南洛阳龙门唐安国相王李旦孺人唐氏墓	Luolong District, Luoyang, Henan	Tang	Mural of a Hu man leading a silk-laden camel	[113]
Hancheng	内蒙古巴林左旗辽上京汉城遗址	Balin Left Banner, Chifeng, Inner Mongolia	Liao	Fragment of a stone camel sculpture	[114]
Kulun Banner Liao Tomb M1	内蒙古库伦旗辽墓M1	Kulun Banner, Tongliao, Inner Mongolia	Liao	Mural with camel-cart procession	[115]
Lamagou Liao Tomb	内蒙古敖汉旗喇嘛沟辽墓	Aohan Banner, Chifeng, Inner Mongolia	Liao	Mural with a camel leader	[116]
Dongfengli Liao Tomb	山西大同东风里辽墓	Pingcheng District, Datong, Shanxi	Liao	Mural with camel	[117]

Yuanbaoshan Yuan Tomb	内蒙古赤峰元宝山元墓	Yuanbaoshan District, Chifeng, Inner Mongolia	Yuan	Mural with camel	[118]
Fujiatun Yuan Tomb	辽宁凌源富家屯元墓	Lingyuan, Chaoyang, Liaoning	Yuan	Mural with camel	[119]
Yidou Northern Qi stone-chamber tomb	山东益都北齐石室墓	Qingzhou, Shandong	Northern Qi	Incised stone image of camel transporting goods	[120]
Tomb of Lou Rui	山西太原北齐娄睿墓	Jinyuan District, Taiyuan, Shanxi	Northern Qi	Mural with camel and a Hu caravan	[121]
Xi'an southern suburbs M31 Tomb	西安南郊 M31 唐墓	Yanta District, Xi'an, Shaanxi	Tang	*Sancai*-glazed camel figurine with a beast-head pannier ornament	[122]
Liuhequwan Jin Tomb	陕西甘泉柳河渠湾金墓	Ganquan County, Yan'an, Shaanxi	Jin	Camel carved on brick	[123]
Ma Village Jin Tomb M4	山西稷山马村金墓 M4	Jishan County, Yun Cheng, Shanxi	Jin	Camel carved on brick	[124]
Qiaocun Cemetery M4309	山西侯马乔村墓地 M4309	Houma village, Lin Fen, Shanxi	Jin	Camel carved on brick	[125]

Sidi Jin Tomb	山西闻喜寺底金墓	Wenxi County, Yuncheng, Shanxi	Jin	Camel carved on brick	[126]
Tomb of Shi Zhe	山西长子县石哲金墓	Zhangzi County, Changzhi, Shanxi	Jin	Camel carved on brick	[127]
Qinshui County Song Tomb	山西沁水县宋墓	Qinshui County, Jincheng, Shanxi	Song	Camel carved on brick	[128]
Mausoleum No. 3 of the Western Xia Imperial Tombs	宁夏银川西夏陵 3 号陵	Xixia District, Yinchuan, Ningxia	Western Xia	Stone camel sculpture	[129]
Yuan Shangdu	内蒙古正蓝旗元上都遗址	Zhenglan Banner, Xilin Gol League, Inner Mongolia	Yuan	White marble camel figurine	[130]
Yangcan Ming Tomb	贵州遵义明播州土司杨粲墓	Zun Yi, Guizhou	Ming	Stone camel sculpture	[131]
Jingheyuan Song Tomb	宁夏泾源县泾河源宋墓	Jingyuan County, Guyuan, Ningxia	Song	Camel carved on brick	[132]
Chuzhou Museum (collection)	江苏淮安楚州博物馆藏	Huai'an District, Huai'an, Jiangsu	Tang	Gilt-bronze camel paperweight	[133]

Xiling Seal Society, Spring Auction 2017 (collection)	西泠印社 2017 年春拍	Hang Zhou, Zhejiang	Qing	Two Qing bronze camel paperweights	[134]
Beijing Zhonghan Auction, Spring Auction 2019 (collection)	北京中汉拍卖 2019 年春拍	Beijing	Qing	Qing gilt-bronze camel paperweight	[135]
Former collection of Kyukyodo, Japan	日本鸠居堂旧藏	Tokyo, Japan (former collection)	Qing	Qing bronze camel paperweight	[136]
Tianjin Cultural Relics Company, Spring Auction 2004 (collection)	天津文物公司 2004 年春拍	Tianjin	Qing	Qing bronze ornament of a camel and herder boy	[137]
Shaanxi History Museum (collection)	陕西历史博物馆藏	Xi'an, Shaanxi	Tang	Bronze mirror with camel motif	[138]
Luoyang Museum (collection)	洛阳博物馆藏	Luoyang, Henan	Tang	Bronze mirror with camel motif	[139]
National Palace Museum, Taipei (collection)	台北故宫博物院藏	Shilin District, Taipei	Han	Bronze seal with camel-shaped knob	[140]
Shanghai Museum (collection)	上海博物馆藏	Huangpu District, Shanghai	Han	Seal with camel-shaped knob	[141]

Luoyang Museum (collection)	洛阳博物馆藏	Luoyang, Henan	Tang	*Sancai*-glazed camel figurine	[142]
Luoyang southern suburbs Tang Tomb	河南洛阳南郊唐墓	Luoyang, Henan	Tang	*Sancai*-glazed camel figurine and camel-leader figurine	[143]
Zhucang Northern Wei Tomb	河南洛阳孟津朱仓北魏墓	Mengjin District, Luoyang, Henan	Northern Wei	Pottery camel figurine	[144]
Liangzuo Northern Wei Tomb	河南洛阳偃师两座北魏墓	Yanshi District, Luoyang, Henan	Northern Wei	Pottery camel figurine	[145]
Hudong Northern Wei Tomb M11	山西大同湖东北魏墓 M11	Yunzhou District, Datong, Shanxi	Northern Wei	Pottery camel figurine	[146]
Tomb of Xianyu Tinghui	陕西西安西郊南何村鲜于庭诲墓	Lianhu District, Xi'an, Shaanxi	Tang, Kaiyuan 11 (723 CE)	*Sancai*-glazed pottery figurine of camel carrying musicians	[56]
Maopo Village M 21	陕西西安长安区茅坡村 M21	Chang'an District, Xi'an, Shaanxi	Sui	Camel figurine	[147]
Joint tomb of Zhang Lin and his wife	陕西西安隋张綝夫妇合葬墓	Yanta District, Xi'an, Shaanxi	Sui, Daye 3 (607 CE)	Camel figurine	[55]
Luoyang southern suburbs Tang Tomb	河南洛阳南郊唐墓	Luolong District, Luoyang, Henan	Tang	*Sancai*-glazed camel figurine and camel-leader figurine	[143]

Luoyang southern suburbs Tang Tomb	河南洛阳南郊唐墓	Luolong District, Luoyang, Henan	Tang	*Sancai*-glazed camel figurine	[148]
Luoyang southern suburbs Tang Tomb	河南洛阳南郊唐墓	Luolong District, Luoyang, Henan	Tang	*Sancai*-glazed figurine of a Hu camel leader	[148]
Luoyang Tang Tomb	河南洛阳唐墓（台北历史博物馆藏）	Luoyang, Henan	Tang	*Sancai*-glazed camel figurine	[149]
Tomb of Mu Tai	甘肃庆城唐穆泰墓	Qingcheng County, Qingyang, Gansu	Tang	Painted camel figurine	[150]
Tomb of Hulu Che	山西太原沙沟隋代斛律彻墓	Jinyuan District, Taiyuan, Shanxi	Sui	Camel-rider figurine	[151]
Tomb of Hulu Che	山西太原隋代斛律彻墓	Jinyuan District, Taiyuan, Shanxi	Sui	Group of pottery figurines (over 300 pieces, including many Hu figurines)	[151]
Tomb of Yuwen Jue	陕西咸阳北周宇文觉墓	Weicheng District, Xianyang, Shaanxi	Northern Zhou	Figurine of camel carrying goods	[152]
Tomb of Lou Rui	山西太原北齐娄睿墓	Jinyuan District, Taiyuan, Shanxi	Northern Qi	Pottery camel figurine	[121]
Tomb of Lü Sili	陕西西安隋吕思礼	Xi'an, Shaanxi	Sui	Pottery camel figurine	[153]

	墓				
Mausoleum of the First Emperor Qin Shi Huang	陕西西安临潼秦始皇帝陵	Lintong District, Xi'an, Shaanxi	Qin	Gold camel statuette	[154]
Mausoleum of the First Emperor Qin Shi Huang	陕西西安临潼秦始皇帝陵	Lintong District, Xi'an, Shaanxi	Qin	Silver camel statuette	[154]
Fujia Northern Qi Stone Chamber Tomb	山东青州傅家北齐石室墓	Qingzhou, Shandong	Northern Qi	Incised stone image of camel transporting goods	[120]
Dayeyu hoard	河北宽城大野峪窖藏	Kuancheng County, Chengde, Hebei	Tang	Silver camel figurines	[122]
Lijiayingzi	内蒙古敖汉旗李家营子墓葬	Aohan Banner, Chifeng, Inner Mongolia	Tang	Silver camel figurines	[122]
Huangye *sancai* kiln	河南巩义黄冶三彩窑址	Gongyi, Zhengzhou, Henan	Tang	*Sancai*-glazed Hu ewer with camel	[122]
Rurilangka	西藏日土县鲁日朗卡岩画	Rutog County, Ngari Prefecture, Tibet	Tang	Rock art with camels	[155]
Ha'erjiao Pasture Kezierkula	新疆吉木乃县哈尔	Jeminay County,	More than 2,000 years	Rock art with a dromedary	[156]

	交牧场克孜尔尔库拉岩画	Altay Prefecture, Xinjiang	ago		
Kulun Banner Liao TombM6	内蒙古库伦旗辽墓M6	Kulun Banner, Tongliao, Inner Mongolia	Liao	Mural with monkey riding a camel	[157]
Inner Mongolia Kulun Banner Liao TombM7	内蒙古库伦旗辽墓M7	Kulun Banner, Tongliao, Inner Mongolia	Liao	Mural with camel carrying goods	[157]
Sijiazi Liao Tomb	辽宁阜新四家子辽墓	Fuxin, Liaoning	Liao	Mural with camel	[157]
Guanshan Liao Tomb M8	辽宁阜新关山辽墓M8	Fuxin, Liaoning	Liao	Mural with camel	[157]
Tomb of Lady Tang, consort of Prince Xiang Li Dan of Anguo	河南洛阳龙门唐安国相王李旦孺人唐氏墓	Luolong District, Luoyang, Henan	Tang	Mural with a Hu merchant leading a camel loaded with silk and hemp bundles	[158]
Tomb of Lady Tang, consort of Prince Xiang Li Dan of Anguo	河南洛阳龙门唐安国相王李旦孺人唐	Luolong District, Luoyang, Henan	Tang	Mural with a Hu figure leading a silk-laden camel	[159]

	氏墓				
Tomb of Lady Tang, consort of Prince Xiang Li Dan of Anguo	河南洛阳龙门唐安国相王李旦孺人唐氏墓	Luolong District, Luoyang, Henan	Tang	Mural with a Hu figure leading a silk-laden camel	[159]
Tomb of Cavalry Commandant Han Yinzhou	甘肃山丹一中唐武骑尉韩胤胄墓	Shandan County, Zhangye, Gansu	Tang	Mold-impressed brick with a Hu merchant leading a camel	[160]
Foyemiaowan Tang Tomb M95	甘肃敦煌佛爷庙湾唐墓（95 号墓）	Dunhuang, Jiuquan, Gansu	Tang	Mold-impressed brick with a Hu figure leading a camel	[161]
Tomb of Qibi Ming	陕西咸阳药王洞村唐契苾明墓	Weicheng District, Xianyang, Shaanxi	Tang	*Sancai*-glazed figurine of a Hu figure leading a dromedary	[162]
Tomb of Qibi Ming	陕西咸阳药王洞村唐契苾明墓	Weicheng District, Xianyang, Shaanxi	Tang	Yellow-glazed figurine of a Hu figure leading a camel	[162]
Tomb of Qibi Ming	陕西咸阳药王洞村唐契苾明墓	Weicheng District, Xianyang, Shaanxi	Tang	Green-glazed camel figurine	[162]
Tomb of Qibi Ming	陕西咸阳药王洞村唐契苾明墓	Weicheng District, Xianyang, Shaanxi	Tang	*Sancai* pagoda-shaped jar with 'Four Filial Exemplars' motif and camel	[162]
Mausoleum of Emperor	陕西富平唐元陵	Fuping County,	Tang	High-relief ostrich stone carving with camel	[163]

Daizong Li Yu	（代宗李豫陵）	Weinan, Shaanxi			
Xuewei Tomb No. 1	青海都兰血渭一号墓	Dulan County, Haixi Prefecture, Qinghai	Tubo period (ca. mid-8th century)	Silver seal with camel motif	[164]
Guanlin Tang Tomb (C7M5657)	河南洛阳关林唐墓（C7M5657）	Luolong District, Luoyang, Henan	Tang	Camel figurine	[165]
Luanshitan Tang Tomb	山西太原乱石滩唐墓	Jinyuan District, Taiyuan, Shanxi	Tang	Mural with camels and horses	[166]
Foyemiaowan Tang Tomb M123	甘肃敦煌佛爷庙湾唐墓 M123	Dunhuang, Gansu	Tang	Mold-impressed brick with a Hu merchant leading a camel	[167]
Shimaping Tang Tomb	甘肃天水市石马坪唐墓	Tianshui, Gansu	Tang	Brick with a Hu man leading a camel	[168]
Changling Machinery Factory Song Tomb	陕西宝鸡长岭机器厂宋墓	Baoji, Shaanxi	Northern Song	Brick with a Hu man leading a camel	[169]
Miaoqu Song Tomb	甘肃镇原庙渠宋墓	Zhenyuan County, Qingyang, Gansu	Northern Song	Brick with a Hu man leading a camel	[170]
Laowanzhuang Jin Tomb	河南焦作老万庄金墓	Jiaozuo, Henan	Jin	Camel motif on stone	[171]

Zhihe Village Song Tomb	四川成都青龙乡致和村宋墓	Chengdu, Sichuan	Southern Song	Brick with camel motif	[172]
Dashata Western Xia Tomb	内蒙古准格尔旗大沙塔西夏墓	Jungar Banner, Ordos, Inner Mongolia	Western Xia	Brick with camel motif	[173]
Spirit Way of the Xiaoling Mausoleum	江苏南京明孝陵神道	Xuanwu District, Nanjing, Jiangsu	Ming	Camel stone sculpture	[174]
Spirit Way of the Thirteen Tombs	北京昌平明十三陵神道	Changping District, Beijing	Ming	Camel stone sculpture	[175]
Jingtai Mausoleum	北京海淀区明景泰陵	Haidian District, Beijing	Ming	Camel stone sculpture	[176]
Spirit Way of the Xianling Mausoleum	湖北钟祥明显陵神道	Zhongxiang, Hubei	Ming	Camel stone sculpture	[177]
Spirit Way of the Tomb of Mu Ying	江苏南京沐英墓神道	Jiangning District, Nanjing, Jiangsu	Ming	Camel stone sculpture	[178]
Spirit Way of the Tomb of Xu Da	江苏南京徐达墓神道	Xuanwu District, Nanjing, Jiangsu	Ming	Camel stone sculpture	[178]

Spirit Way of the Tomb of Li Wenzhong	江苏南京李文忠墓神道	Xuanwu District, Nanjing, Jiangsu	Ming	Camel stone sculpture	[178]
Ming Imperial Mausoleum,	安徽凤阳明皇陵	Fengyang County, Chuzhou, Anhui	Ming	Camel stone sculpture	[179]
Spirit Way of the Tomb of Prince Xiangjian	湖北襄阳明襄简王墓神道	Xiangyang, Hubei	Ming	Camel stone sculpture	[180]
Spirit Way of the Tomb of Prince Lujian	河南新乡潞简王墓神道	Xinxiang, Henan	Ming	Camel stone sculpture	[181]
Changling Machinery Factory Song Tomb	陕西宝鸡长岭机器厂宋墓	Weibin District, Baoji, Shaanxi	Northern Song	Brick with a Hu man leading a camel	[182]
Laowanzhuang Jin Tomb	河南焦作老万庄金墓	Jiaozuo, Henan	Jin	Camel motif on stone	[171]
Dashata Western Xia Tomb	内蒙古准格尔旗大沙塔西夏墓	Jungar Banner, Ordos, Inner Mongolia	Western Xia	Brick with camel motif	[173]
Spirit Way of the Xiaoling Mausoleum	江苏南京明孝陵神道	Xuanwu District, Nanjing, Jiangsu	Ming	Camel stone sculpture	[174]

Spirit Way of the Thirteen Tombs	北京昌平明十三陵神道	Changping District, Beijing	Ming	Camel stone sculpture	[175]
Spirit Way of the Xianling Mausoleum	湖北钟祥明显陵神道	Zhongxiang, Hubei	Ming	Camel stone sculpture	[177]
Ming Imperial Mausoleum,	安徽凤阳明皇陵	Fengyang County, Chuzhou, Anhui	Ming	Camel stone sculpture	[179]
Spirit Way of the Tomb of Prince Lujian	河南新乡潞简王墓神道	Fengquan District, Xinxiang, Henan	Ming	Camel stone sculpture	[181]
Chahantemaitu	青海德令哈察汗特买图岩画	Delingha, Haixi Prefecture, Qinghai	Sui-Tang	Rock art with camel and hunting scene	[183]
Chahantemaitu	青海德令哈察汗特买图岩画	Delingha, Haixi Prefecture, Qinghai	Sui-Tang	Rock art with camel and tree	[183]
Da'aobao	内蒙古包头固阳县大敖包山岩画	Guyang County, Baotou, Inner Mongolia	Northern Dynasties-Sui-Tang	Rock art with camels, sheep, and horses	[184]
Jirukenzhadegai	内蒙古阿拉善左旗吉如肯札德盖岩画	Alxa Left Banner, Inner Mongolia	Tang-Yuan	Rock art with ridden a camel	[185]

Jirukenzhadegai	内蒙古阿拉善左旗吉如肯札德盖岩画	Alxa Left Banner, Inner Mongolia	Tang-Yuan	Rock art with camels and hunting scene	[185]
Mandela Mountain	内蒙古阿拉善右旗曼德拉山岩画	Alxa Right Banner, Inner Mongolia	Tang-Western Xia	Rock art with camel herd	[186]
Mandela Mountain	内蒙古阿拉善右旗曼德拉山岩画	Alxa Right Banner, Inner Mongolia	Tang-Western Xia	Rock art with camel rider	[186]
Hanwula Mountain	内蒙古乌拉特中旗韩乌拉山岩画	Urad Middle Banner, Bayannur, Inner Mongolia	Tang	Rock art with camel and herder	[187]
Qincheng Zheyaogou	新疆哈密市沁城折腰沟岩画	Yizhou District, Hami, Xinjiang	Tang	Rock art with camel	[188]
Bostan Pasture	新疆木垒县博斯坦牧场岩画	Mulei County, Changji Prefecture, Xinjiang	Tang	Rock art with camel and rider	[189]
Heishan	甘肃嘉峪关黑山岩画	Jiayuguan, Gansu	Tang	Rock art with camel	[190]
Daheigou	甘肃肃北蒙古族自	Subei County,	Tang	Rock art with camel herd	[191]

	治县大黑沟岩画	Jiuquan, Gansu			
Halong	青海刚察县哈龙	Gangca County, Haibei Prefecture, Qinghai	Tang	Rock art with camels and yaks	[192]
Lushan	青海天峻县卢山	Tianjun County, Haixi Prefecture, Qinghai	Tang	Rock art with camel rider	[193]
HelanKou	宁夏贺兰山贺兰口	Helan County, Yinchuan, Ningxia	Tang-Western Xia	Rock art with camel	[194]
Damaidi	宁夏中卫大麦地	Zhongwei, Ningxia	Tang	Rock art with camel and rider	[195]
Renmudong	西藏日土县任姆栋	Rutog County, Ngari Prefecture, Tibet	Tang	Rock art with camels and hunting scene	[196]

References

[1] Anhui Provincial Institute of Cultural Relics and Archaeology, Chaohu City Cultural Relics Management Office. *Chaohu Han Tombs*; Cultural Relics Press: Beijing, China, 2007; p. 34. (In Chinese)

[2] a. Anhui Provincial Cultural Relics Team, Fuyang Prefecture Museum, Fuyang Cultural Bureau. Excavation Brief of the Western Han Ruyinhou Tomb at Shuanggudui, Fuyang. *Cult. Relics* **1978**, *8*, 12-15. (In Chinese) b. Lu, Y.; Shan, Y. Animal Motif Belt Plaques Unearthed from Western Han Tombs. *Archaeol. Cult. Relics* **2007**, *4*, 38-45. (In Chinese)

[3] Liu, Y. Ancient Official Seals Unearthed in Jingchuan, Gansu. *Archaeol. Cult. Relics* **1988**, *1*, 25-30. (In Chinese)
[4] Wang, C.F., Ed. *Corridor of the Silk Road: A Selection of Gansu Cultural Relics*; Beijing Times Chinese Press: Beijing, China, 2020. (In Chinese)
[5] Chen, J. *The Sorting and Study of the Warring States Period Vehicle Ornaments in the Collection of Tianshui Museum. Master's Thesis, Northwest Normal University, Lanzhou, China, 2020*; p. 14. (In Chinese)
[6] a. Han Pingling Archaeological Team. Giant Animals Buried with the Young Emperor. *Cult. Relics World* **2002**, *1*, 10-15. (In Chinese) b. Yuan, J. New Discoveries and Progress in Zooarchaeological Research. *Archaeology* **2004**, *7*, 54-60. (In Chinese) c. Pang, B. A large number of camel bones excavated from the burial pits of Emperor Zhao. China Cultural Relics News, 7 December 2001, p. 1. (In Chinese)
[7] Institute of Archaeology, Chinese Academy of Social Sciences, et al. *Excavation Report of the Mancheng Han Tombs*; Cultural Relics Press: Beijing, China, 1980; pp. 253-256. (In Chinese)
[8] a. Hu, J. The Kings of Zhongshan and Their Tombs in the Western and Eastern Han Dynasties. *Cult. Relics Spring Autumn* **2000**, *1*, 30-35. (In Chinese) b. *Hebei Provincial Cultural Relics Management Office. Thirty Years of Cultural Relics and Archaeological Work (1949-1979)*; Cultural Relics Press: Beijing, China, 1979; pp. 35-53. (In Chinese)
[9] Chen, P. *Research on the Northern Youyan Culture*; Qunyan Press: Beijing, China, 2006; p. 362. (In Chinese)
[10] Luoyang Second Cultural Relics Team, Department of History of Science and Technology and Archaeometry, University of Science and Technology of China. Discovery of a "Xiongnu Guihan Jun" Bronze Seal in a Donghan Tomb at Xindian, Luoyang. *Cult. Relics* **2003**, *9*, 55-60. (In Chinese)
[11] Henan Provincial Institute of Cultural Relics and Archaeology, Yongcheng City Cultural Relics and Tourism Administration. *Han Tombs at Huangtushan and Cuocheng in Yongcheng*; Elephant Press: Zhengzhou, China, 2010; Color Plate 35. (In Chinese)
[12] Hubei Provincial Cultural Bureau Cultural Relics Team. A Large Number of Important Cultural Relics Unearthed from Three Large Chu Tombs in Jiangling, Hubei. *Cult. Relics* **1966**, *5*, 1-10. (In Chinese)
[13] Chen, Z. Discovery of Camel Images from the Warring States and Western Han Periods in Hubei. *Agric. Archaeol.* **1987**, *1*, 200-205. (In Chinese)
[14] Hunan Provincial Museum. Brief Report on the Excavation of Han Tomb No. 304 at Yangjiashan, Changsha. In *Collected Papers of Archaeology (1)*; China Social Sciences Press: Beijing, China, 1981; pp. 150-155. (In Chinese)
[15] Ge, C. Heavenly Horses and Camels: A New Interpretation of Symbolic Markers of the Silk Road in the Han Dynasty. *Palace Mus. J.* **2018**, *1*, 6-20. (In Chinese)
[16] a. Hubei Provincial Cultural Bureau Cultural Relics Team. A Large Number of Important Cultural Relics Unearthed from Three Large Chu Tombs in Jiangling, Hubei. *Cult. Relics* **1966**, *5*, 1-10. (In Chinese) b. Chen, Z. Discovery of Camel Images from the Warring States and Western Han Periods in Hubei. *Agric. Archaeol.* **1987**, *1*, 200-205. (In Chinese) c.Feng, K.; Li, Y.; Jiang, W.; Wang, T. A Preliminary Study on Cultural Relics with Camel Themes from the Eastern Zhou, Qin, and Han Periods. Archaeol.

Cult. Relics 2024, 3, 82-92. (In Chinese)
[17] Li, B. *Chinese National Treasures: A Collection of Precious Cultural Relics from Shaanxi: Gold and Silver Ware Volume*; Shaanxi People's Education Press: Xi'an, China, 1998; pp. 186-187. (In Chinese)
[18] a. *Ordos Museum. Ordos Bronzes*; Cultural Relics Press: Beijing, China, 2006; p. 112. (In Chinese) b. *Emperor Qinshihuang's Mausoleum Site Museum. Germination, Growth, Fusion: A Collection of Northern Bronze Culture from the Eastern Zhou Period*; Sanqin Press: Xi'an, China, 2012; p. 135. (In Chinese)
[19] Ordos Bronzes Museum. *Bronze Empire on Horseback*; Science Press: Beijing, China, 2021; p. 127. (In Chinese)
[20] Ordos Bronzes Museum. *Bronze Empire on Horseback*; Science Press: Beijing, China, 2021; p. 76. (In Chinese)
[21] a. *Li, W. Rongdi and Xiongnu Bronze Culture: Civilization of the Grassland Silk Road*; Cultural Relics Press: Beijing, China, 2017; pp. 156-157. (In Chinese) b. *Ordos Museum. Ordos Bronzes*; Cultural Relics Press: Beijing, China, 2006; pp. 166, 169, 170, 183, 187, 235. (In Chinese)
[22] a. Lu, S. Three Han Dynasty Official Seals Unearthed in Inner Mongolia's Yikezhao League. *Cult. Relics* **1977**, *5*, 85-88. (In Chinese) b. *Liu, W., Ed. Fax of Chinese Civilization: Forging the Imperial Way (Qin and Han)*; Shanghai Lexicographical Publishing House: Shanghai, China, 2001; p. 95. (In Chinese)
[23] Ningxia Hui Autonomous Region Institute of Cultural Relics and Archaeology. Excavation Brief of a Western Han Tomb (IIIM3) in the Suburbs of Guyuan City, Ningxia. *Cult. Relics* **2023**, *7*, 4-15. (In Chinese)
[24] Ningxia Hui Autonomous Region Institute of Cultural Relics and Archaeology, Pengyang County Cultural Relics Station. Spring and Autumn and Warring States Period Cemetery at Zhangjie Village, Pengyang County, Ningxia. *Archaeology* **2002**, *8*, 15-30. (In Chinese)
[25] Wu, E.; Zhong, K.; Li, J. The Xiongnu Cemetery at Daodunzi, Tongxin, Ningxia. *Acta Archaeol. Sin.* **1988**, *3*, 333-356. (In Chinese)
[26] Emperor Qinshihuang's Mausoleum Site Museum. New Progress in the Archaeology of the Mausoleum of the First Qin Emperor: Important Achievements in the Exploration and Excavation of Tombs to the West of the Mausoleum. China Cult. Relics News 2020, June 19, 5. (In Chinese)
[27] Qinghai Provincial Cultural Relics Management Office Archaeological Team. A Xiongnu Tomb at Shang Sunjiazhai in Datong, Qinghai. *Cult. Relics* **1979**, *4*, 49-53. (In Chinese)
[28] Zheng, Y.; Jia, D. Han Dynasty Bronze Camel-shaped Weights. *Cult. Relics World* **1993**, *6*, 40-41. (In Chinese)
[29] Zibo Municipal Museum, Shandong Province. Pits of Burial Objects for the Western Han King of Qi's Tomb. *Acta Archaeol. Sin.* **1985**, *2*, 223-266. (In Chinese)
[30] a. Li, C. Recording the Hu Fu Wine-Warming Zun Unearthed in Youyu County. *Cult. Relics World* **2008**, *3*, 60-62. (In Chinese) b. Chen, L. From Feathered Man to Queen Mother of the West: A Study of the Images on the Hu Fu Wine-Warming Zun. *Northwest Fine Arts* **2020**, *2*, 120-125. (In Chinese)
[31] UNESCO Office in Beijing, Xinjiang Bureau of Cultural Heritage, Xinjiang Institute of Cultural Relics and Archaeology. *Jiaohe Ruined City:* **1993**, *1994* Annual Archaeological Excavation Report; Oriental Press: Beijing, China, 1998; pp. 36-44, 64. (In Chinese)

[32] Yu, M. *Complete Collection of Chinese Art: Arts and Crafts Volume (Part 1)*; Qinghai People's Publishing House: Xining, China, 2003; p. 187. (In Chinese)
[33] a. *Xi'an Museum. Xi'an Museum (Illustrated Catalogue)*; World Publishing Corporation: Beijing, China, 2007; p. 25. (In Chinese) b. Song, Y. Research on Glazed Pottery Toy Figurines of the Han Dynasty: A Case Study of Miniature Glazed Pottery Figurines Unearthed in Xi'an and Luoyang Areas. *Cult. Relics Cent. China* **2023**, *5*, 90-98. (In Chinese)
[34] Wang, L. The Elegance of Grassland National Cultural Relics: A Tour of the Altay National Cultural Relics Exhibition. *Xinjiang Cult. Relics* **1994**, *2*, 85-88. (In Chinese)
[35] a. Jia, Y. A Brief Discussion on the Wool Textiles Unearthed from the Niya Site. *Cult. Relics* **1980**, *3*, 70-75. (In Chinese) b. *Yu, Y. Study on Woolen Garments and Textiles Unearthed from the Shanpula Cemetery. Master's Thesis, Donghua University, Shanghai, China, 2010*; pp. 71-91. (In Chinese) c. Chen, X. The Early Diffusion of Camels in the Western Regions from Archaeological Materials. In *Cultural Relics, Documents and Culture: Collection of Essays by Young Historical Archaeologists (Vol. 1)*; Shanghai Classics Publishing House: Shanghai, China, 2017; pp. 200-215. (In Chinese)
[36] Zhang, J. *The Development and Evolution of Khotan Culture in the Han and Jin Dynasties.* Ph.D. Thesis, Peking University, Beijing, China, 2007; pp. 19-23. (In Chinese)
[37] Xinjiang Uygur Autonomous Region Museum, Bayingolin Mongol Autonomous Prefecture Cultural Relics Management Office, Qiemo County Cultural Relics Management Office. Excavation Report of Cemetery No. 1 at Zhagunluke, Qiemo, Xinjiang. *Acta Archaeol. Sin.* **2003**, *1*, 89-120. (In Chinese)
[38] Xinjiang Uygur Autonomous Region Bureau of Cultural Heritage. *A Grand View of Xinjiang Cultural Relics and Historic Sites*; Xinjiang Art and Photography Press: Urumqi, China, 1999; p. 230. (In Chinese)
[39] Datong Museum & Shanxi Provincial Cultural Relics Work Committee. The Northern Wei Tomb of Sima Jinlong at Shijiazhai, Datong, Shanxi. *Cult. Relics* **1972**, (3). (In Chinese)
[40] Shanxi Provincial Institute of Archaeology & Datong Municipal Institute of Archaeology. Excavation of a Northern Wei Tomb at Chenzhuang, Datong County, Datong City, Shanxi. *Cult. Relics* **2011**, (12). (In Chinese)
[41] Datong Municipal Institute of Archaeology. Excavation of a Northern Wei Tomb with Murals at Wenyinglu, Datong, Shanxi. *Cult. Relics* **2011**, (12). (In Chinese)
[42] Li, H. P., et al. Excavation of Two Northern Wei Tombs in Yanshi, Luoyang. *Cult. Relics Cent. China* **2019**, (6). (In Chinese)
[43] Wang, W. H., & Wang, Q. Y. Excavation of Northern Wei Tomb HM555 on Shachang West Road, Luoyang. *Cult. Relics* **2002**, (9). (In Chinese)
[44] Yan, H., et al. Excavation of a Northern Wei Tomb on Hengshan Road, Jianxi, Luoyang. *Cult. Relics* **2016**, (7). (In Chinese)
[45] Cheng, Y. J. Excavation of Two Northern Wei Tombs in Jili District, Luoyang, Henan. *Archaeology* **2011**, (9). (In Chinese)
[46] Ningxia Institute of Cultural Relics and Archaeology. Excavation of the Northern Wei Tomb of Li Xian and His Wife. *Cult. Relics* **1985**, (11). (In Chinese)
[47] Shaanxi Provincial Museum. Excavation of a Tang Dynasty Tomb at Zhongbaocun in the Western Suburbs of Xi'an. *Archaeology* **1960**, (3), 34-38. (In Chinese)

[48] Xi'an Institute of Cultural Relics Preservation. (2004). Excavation of Tang Tomb M31 in the Southern Suburbs of Xi'an. *Archaeol. Cult. Relics* **2004**, (3), 12-18. / Ran, W. L. (2017). A Three-Color Glazed Camel Figurine from the Perspective of the Silk Road. *Tang Hist. Rev.* **2017**, *24*, 156-169. (In Chinese)
[49] Shang, M. (2018). *A Study on the Plastic Art of Camel Figurines Carrying Musicians Unearthed from Tang Tombs* (Master's thesis). Xi'an Academy of Fine Arts, 2018, pp. 23-25. (In Chinese)
[50] Xi'an Institute of Cultural Relics Preservation. Excavation of Sui and Tang Tombs at the Thermal Power Plant Construction Site in the Western Suburbs of Xi'an. *Archaeol. Cult. Relics* **1991**, (4), 45-52. (In Chinese)
[51] Zhang, Q. M. A Three-Color Glazed Camel Unearthed from a Tang Tomb in the Eastern Suburbs of Xi'an. *Cult. Relics World* **1996**, (3), 34-36. (In Chinese)
[52] Shaanxi Provincial Institute of Archaeology, et al. (2025). *Excavation Report on the Tomb of Tang Chancellor Han Xiu and His Wife*. Beijing: Science Press, 2025, pp. 156-160. (In Chinese)
[53] Li, Y. S., & Tian, Y. Q. A Preliminary Study on the Molded Images on the Camel Figurine's Pack Saddle from a Sui Dynasty Tomb in Maopo Village, Xi'an. *Archaeol. Cult. Relics* **2018**, (3), 51-58. (In Chinese)
[54] Duan, Y., et al. Excavation of Sui and Tang Tombs at Beizhu Village, Beidu Town, Xianyang, Shaanxi. *J. Natl. Mus. China* **2023**, (8), 34-41. (In Chinese)
[55] Xi'an Institute of Cultural Relics Preservation. Excavation of the Sui Dynasty Tomb of Zhang Chen and His Wife in Xi'an. *Cult. Relics* **2004**, (1), 45-52. (In Chinese)
[56] Institute of Archaeology, Chinese Academy of Social Sciences. (1980). *Sui and Tang Tombs in the Suburbs of Tang Chang'an*. Beijing: Cultural Relics Press, 1980, pp. 67-72. (In Chinese)
[57] Shaanxi Provincial Museum. Excavation of the Tang Tomb of Zheng Rentai. *Cult. Relics* **1972**, (7), 33-42. (In Chinese)
[58] Shaanxi Provincial Museum. Excavation of the Tang Tomb of Crown Prince Yide. *Cult. Relics* **1972**, (7), 26-32. (In Chinese)
[59] Shaanxi Provincial Museum. Excavation of the Tang Tomb of Crown Prince Zhanghuai. *Cult. Relics* **1972**, (7), 13-25. (In Chinese)
[60] Fuping County Cultural Center. Excavation of the Tang Tomb of Li Feng. *Archaeology* **1977**, (5), 313-318. (In Chinese)
[61] Shaanxi Provincial Institute of Archaeology. Excavation of a Tang Tomb at Nanliwang Village, Chang'an County. *Wenbo* **1990**, (3), 23-29. (In Chinese)
[62] Shanxi Provincial Institute of Archaeology. Excavation of the Sui Dynasty Tomb of Hulü Che in Taiyuan. *Cult. Relics* **1992**, (10), 12-19. (In Chinese)
[63] Shanxi Provincial Institute of Archaeology. Excavation of the Sui Dynasty Tomb of Yu Hong in Taiyuan. *Cult. Relics* **2001**, (1), 27-43. (In Chinese)
[64] Hou, X. G., et al. Excavation of a Tang Dynasty Dated Brick-Chambered Tomb at Zhijiabao, Datong, Shanxi. *J. Natl. Mus. China* **2023**, (8), 42-49. (In Chinese)
[65] Yang, Z. S. (2023). The Unique Style of Shangdang: A Study of Tang Dynasty Hu Merchant Riding Camel Figurines from Changzhi, Shanxi. *The Paper*, 2023-12-11. (In Chinese)
[66] Changzhi Municipal Museum. Excavation of a Tang Dynasty Tomb in Changzhi, Shanxi. *Cult. Relics* **2004**, (8), 34-40. (In Chinese)

[67] Xingtai Prefectural Office for the Preservation of Cultural Relics. The Tang Dynasty Tomb of Guo Xiang in Nanhe, Hebei. *Cult. Relics* **1993**, (6), 23-29. (In Chinese)
[68] Cheng, Y. J. & Zhou, L. (Eds.). (2017). *The Tang Dynasty Tomb of An Pu and His Wife at Longmen, Luoyang*. Beijing: Science Press, 2017. (In Chinese)
[69] Cixian County Cultural Center. Excavation of the Eastern Wei Tomb of Princess Ruru in Cixian, Hebei. *Cult. Relics* **1984**, (4), 1-9. (In Chinese)
[70] Institute of Archaeology, Chinese Academy of Social Sciences. (2003). *The Northern Dynasties Mural Tomb at Wanzhang, Cixian*. Beijing: Science Press, 2003, pp. 78-82. (In Chinese)
[71] Beijing Municipal Institute of Cultural Relics. The Tang Tomb of Shi Siming in Fengtai, Beijing. *Cult. Relics* **1991**, (9), 28-35. (In Chinese)
[72] Liaoning Provincial Institute of Cultural Relics and Archaeology. Excavation of a Tang Dynasty Tomb in Chaoyang, Liaoning. *Archaeology* **2004**, (8), 45-52. (In Chinese)
[73] Inner Mongolia Museum. Excavation of a Northern Wei Tomb on University East Road, Hohhot. *Cult. Relics* **1977**, (5), 38-42. (In Chinese)
[74] Xu, C. (2023). *A Study of the Tang Dynasty Tomb at Bayannur in the Northern Desert Grassland*. Beijing: Social Sciences Academic Press, 2023, pp. 89-95. (In Chinese)
[75] Ningxia Institute of Cultural Relics and Archaeology. (2021). *Excavation Report on the Tang Tombs at Nanyuan, Guyuan*. Beijing: Cultural Relics Press, 2021, pp. 123-128. (In Chinese)
[76] Ningxia Institute of Cultural Relics and Archaeology. (2021). *Excavation Report on the Tang Tombs at Nanyuan, Guyuan*. Beijing: Cultural Relics Press, 2021, pp. 156-160. (In Chinese)
[77] Ningxia Institute of Cultural Relics and Archaeology. Excavation of a Tang Dynasty Tomb at Wanglaoba, Guyuan, Ningxia. *Archaeol. Cult. Relics* **2022**, (4), 23-29. (In Chinese)
[78] Ningxia Institute of Cultural Relics and Archaeology. (2006). *Tang Tombs in the Western Suburbs of Wuzhong*. Beijing: Cultural Relics Press, 2006, pp. 67-71. (In Chinese)
[79] Ningxia Institute of Cultural Relics and Archaeology. Excavation of a Tang Tomb in Yanchi, Ningxia. *Cult. Relics* **1988**, (9), 23-30. (In Chinese)
[80] Ningxia Institute of Cultural Relics and Archaeology. (n.d.). Painted Camel Figurine. *Ningxia Inst. Cult. Relics Archaeol. Off. Website*. (In Chinese)
[81] Chen, B. Y. A Study of the Inscriptions and Burial Customs of the Western Xia Tomb in the Western Suburbs of Wuwei, Gansu. *Archaeol. Cult. Relics* **1994**, (3), 45-51. (In Chinese)
[82] Wuwei Prefectural Museum. Excavation of a Western Xia Tomb at Zhangyi, Wuwei, Gansu. *Cult. Relics* **2004**, (6), 34-40. (In Chinese)
[83] Gansu Provincial Museum. The Yuan Dynasty Family Tombs of Wang Shixian in Zhangxian, Gansu. *Cult. Relics* **1982**, (2), 1-12. (In Chinese)
[84] Ningxia Institute of Cultural Relics and Archaeology. (2013). *The Western Xia Imperial Tombs*. Beijing: Cultural Relics Press, 2013, pp. 234-238. (In Chinese)

[85] Ikh Juu League Cultural Relics Workstation. Excavation of a Western Xia Tomb in Ejin Horo Banner, Inner Mongolia. *Archaeology* **1992**, (5), 432-438. (In Chinese)
[86] Xiang, C. S. A Yuan Dynasty Mural Tomb at Sanyanjing, Chifeng, Inner Mongolia. *Cult. Relics* **1982**, (1), 45-51. (In Chinese)
[87] Chifeng Municipal Museum. A Yuan Dynasty Mural Tomb at Shazishan, Chifeng, Inner Mongolia. *Cult. Relics* **1993**, (2), 34-40. (In Chinese)
[88] Institute of Archaeology, Chinese Academy of Sciences. Archaeological Discoveries in the Yuan Dynasty Capital, Dadu. *Archaeology* **1972**, (1), 23-31. (In Chinese)
[89] Wang, J. G. Excavation of the Yuan Dynasty Tomb of Wang Shiying in the Southern Suburbs of Xi'an. *Wenbo* **2005**, (2), 12-18. (In Chinese)
[90] Xi'an Institute of Cultural Relics Preservation. The Yuan Dynasty Tomb of Liu Yishi in the Eastern Suburbs of Xi'an. *Cult. Relics* **2008**, (4), 34-41. (In Chinese)
[91] Datong Municipal Cultural Relics Exhibition Hall. Excavation of the Yuan Dynasty Tombs of Feng Daozhen and Wang Qing in Datong, Shanxi. *Cult. Relics* **1962**, (10), 34-42. (In Chinese)
[92] Shanxi Provincial Cultural Relics Management Committee. An Ancient Tomb at Beiyukou, Wenshui, Shanxi. *Archaeology* **1961**, (3), 136-139. (In Chinese)
[93] Xi'an Institute of Cultural Relics Preservation. Excavation of the Yuan Dynasty Tomb of Zhang Dafu at Qujiang, Xi'an. *Cult. Relics* **2008**, (4), 42-48. (In Chinese)
[94] Nanjing Municipal Museum. Excavation Report on the Accompanying Tombs of the Ming Xiaoling Mausoleum. *Archaeology* **1998**, (8), 56-63. (In Chinese)
[95] Nanjing Municipal Museum. The Tomb of Mu Ying, Prince of Qianning of the Ming Dynasty. *Archaeology* **1999**, (10), 45-52. (In Chinese)
[96] Nanjing Municipal Museum. The Family Tombs of Xu Da, Prince of Zhongshan of the Ming Dynasty. *Nanjing Cult. Relics* **1993**, (2), 23-29. (In Chinese)
[97] Nanjing Municipal Committee for the Preservation of Cultural Relics. Excavation of the Ming Dynasty Tomb of Deng Yu in Nanjing. *Cult. Relics* **1973**, (3), 34-39. (In Chinese)
[98] Institute of Archaeology, Chinese Academy of Social Sciences. (1990). *The Dingling Mausoleum*. Beijing: Cultural Relics Press, 1990, pp. 267-272. (In Chinese)
[99] Shandong Provincial Museum. (2014). *The Tomb of Prince Luhuang*. Beijing: Cultural Relics Press, 2014, pp. 156-160. (In Chinese)
[100] Chengdu Institute of Cultural Relics and Archaeology. (2007). *The Ming Dynasty Shu Princely Tombs in Chengdu*. Beijing: Cultural Relics Press, 2007, pp. 89-94. (In Chinese)
[101] Hubei Provincial Institute of Cultural Relics and Archaeology. (2007). *The Tomb of Prince Liangzhuang*. Beijing: Cultural Relics Press, 2007, pp. 134-138. (In Chinese)
[102] Jiangxi Provincial Museum. The Ming Dynasty Family Tombs of the Prince of Ning in Nanchang. *Cult. Relics* **1998**, (7), 34-41. (In Chinese)
[103] The Palace Museum. (2008). *Compendium of Treasures from the Palace Museum Collection: Jadeware (Vol. 2)*. Beijing: Forbidden City Press, 2008, p. 156. (In Chinese)
[104] The Palace Museum. (2008). *Compendium of Treasures from the Palace Museum Collection: Jadeware (Vol. 2)*. Beijing: Forbidden City Press, 2008, p. 167. (In Chinese)
[105] The Palace Museum. (2004). *Masterpieces of Carving in the Palace Museum Collection*. Beijing: Forbidden City Press, 2004, p. 89. (In Chinese)

[106] Deng, S. P. On the "Jade Figurine Tending a Camel" in the Palace Museum Collection. *Natl. Palace Mus. Mon. Chin. Art* **1987**, (56), 34-41. (In Chinese)
[107] Chengde Municipal Cultural Relics Bureau. (2010). *The Mountain Resort and the Eight Outer Temples*. Beijing: Cultural Relics Press, 2010, pp. 123-126. (In Chinese)
[108] Chengde Municipal Cultural Relics Bureau. (2010). *The Mountain Resort and the Eight Outer Temples*. Beijing: Cultural Relics Press, 2010, pp. 145-147. (In Chinese)
[109] Tibet Administrative Commission of Cultural Heritage. (2005). *Norbulingka*. Beijing: Cultural Relics Press, 2005, pp. 78-81. (In Chinese)
[110] Ordos Museum. (2004). *Collected Essays on Cultural Relics and Archaeology in Ordos*. Hohhot: Yuanfang Press, 2004, pp. 234-237. (In Chinese)
[111] Inner Mongolia Museum. (2002). *Ethnic Cultural Relics of Inner Mongolia*. Beijing: Cultural Relics Press, 2002, pp. 89-92. (In Chinese)
[112] Xinjiang Museum. (1992). *Folk Carpets of Xinjiang*. Urumqi: Xinjiang People's Publishing House, 1992, pp. 56-59. (In Chinese)
[113] Zhang, J. W. (2025). Modern Camel Caravan Retraces Silk Road, Resembling Tang Dynasty Murals Unearthed in Luoyang. *Luoyang Daily*, 2025-10-31. (In Chinese)
[114] Inner Mongolia Institute of Cultural Relics and Archaeology. (1994). Survey Report on the Site of the Upper Capital of Liao Dynasty. In *Collected Papers on Inner Mongolia Cultural Relics and Archaeology (Vol. 1)* (pp. 235-241). Beijing: Encyclopedia of China Publishing House. (In Chinese)
[115] Wang, J. Q., & Chen, X. W. (1989). *Liao Dynasty Mural Tombs in Kulun*. Beijing: Cultural Relics Press, 1989, pp. 45-52. (In Chinese)
[116] Aohan Banner Museum. A Liao Dynasty Mural Tomb at Lamagou in Aohan Banner. *Inner Mongolia Cult. Relics Archaeol.* **1999**, (1), 23-29. (In Chinese)
[117] Datong Municipal Institute of Archaeology. Excavation of a Liao Dynasty Mural Tomb at Dongfengli, Datong, Shanxi. *Cult. Relics* **2013**, (10), 45-52. (In Chinese)
[118] Xiang, C. S. A Yuan Dynasty Mural Tomb at Yuanbaoshan, Chifeng, Inner Mongolia. *Cult. Relics* **1983**, (4), 45-51. (In Chinese)
[119] Liaoning Provincial Museum. A Yuan Dynasty Tomb at Fujiatun, Lingyuan. *Cult. Relics* **1985**, (6), 56-62. (In Chinese)
[120] Xia, M. C. Incised Stone Carvings from a Northern Qi Stone Chamber Tomb in Yidu. *Cult. Relics* **1985**, (10), 45-51. (In Chinese)
[121] Shanxi Provincial Institute of Archaeology. Excavation of the Northern Qi Tomb of Lou Rui in Taiyuan. *Cult. Relics* **1983**, (10), 1-23. (In Chinese)
[122] Ran, W. L. (2017). A Three-Color Glazed Camel Figurine from the Perspective of the Silk Road. *Tang Hist. Rev.* **2017**, *24*, 156-169. (In Chinese)
[123] Ganquan County Museum. Excavation of a Jin Dynasty Tomb in Ganquan, Shaanxi. *Wenbo* **2003**, (5), 12-18. (In Chinese)
[124] Shanxi Provincial Institute of Archaeology. Excavation of Jin Dynasty Tombs in Jishan, Shanxi. *Cult. Relics* **1983**, (1), 45-51. (In Chinese)
[125] Shanxi Provincial Institute of Archaeology. (2004). *Qiaocun Cemetery in Houma (1959-1996)*. Beijing: Cultural Relics Press, 2004, pp. 235-238. (In Chinese)
[126] Shanxi Provincial Institute of Archaeology. A Jin Dynasty Tomb at Sidi, Wenxi, Shanxi. *Cult. Relics* **2010**, (3), 34-41. (In Chinese)
[127] Shanxi Provincial Institute of Archaeology. A Jin Dynasty Tomb with Murals at Shizhe, Zhangzi County, Shanxi. *Cult. Relics* **1985**, (6), 45-52. (In Chinese)
[128] Shanxi Provincial Institute of Archaeology. Excavation of a Song Dynasty Tomb in Qinshui County, Shanxi. *Cult. Relics* **2015**, (7), 28-35. (In Chinese)
[129] Ningxia Institute of Cultural Relics and Archaeology. (2013). *The Western Xia Imperial Tombs*. Beijing: Cultural Relics Press, 2013, pp. 156-160. (In Chinese)
[130] Inner Mongolia Institute of Cultural Relics and Archaeology. (2008). *Xanadu (Shangdu) of the Yuan Dynasty*. Beijing: Encyclopedia of China Publishing House, 2008,

pp. 189-194. (In Chinese)
[131] Guizhou Provincial Museum. (1965). *Excavation Report on the Tomb of Yang Can, Tusi of Bozhou, in Zunyi*. Beijing: Cultural Relics Press, 1965, pp. 34-37. (In Chinese)
[132] Ningxia Museum. (2019). Camel Pattern Brick Carving. *Ningxia Inst. Cult. Relics Archaeol. Off. Website* **2019**. (In Chinese)
[133] Chinese People's Political Consultative Conference Huai'an District Committee. (n.d.). Tang Dynasty Gilt Bronze Camel Paperweight. *CPPCC Huai'an District Comm. Off. Website*. (In Chinese)
[134] Xiling Yinshe Auction Co., Ltd. (2017). Two Qing Dynasty Bronze Camel Paperweights. *Xiling Yinshe Auction Off. Website* **2017**. (In Chinese)
[135] Beijing Zhonghan Auction Co., Ltd. (2019). Qing Dynasty Gilt Bronze Camel Paperweight. *Beijing Zhonghan Auction Off. Website* **2019**. (In Chinese)
[136] Qi, G. (1999). *Collected Essays of Qi Gong*. Beijing: Zhonghua Book Company, 1999, p. 245. (In Chinese)
[137] Tianjin Cultural Relics Company. (2004). *Tianjin Cultural Relics Company 2004 Spring Auction Catalogue*. Tianjin: Tianjin Cultural Relics Company, 2004, p. 78. (In Chinese)
[138] Shaanxi History Museum. (n.d.). Tang Dynasty Auspicious Beast and Grape Pattern Bronze Mirror. *Shaanxi Hist. Mus. Off. Website*. (In Chinese)
[139] Luoyang Museum. (n.d.). Tang Dynasty Sea Beast and Grape Pattern Bronze Mirror. *Luoyang Mus. Off. Website*. (In Chinese)
[140] National Palace Museum. (n.d.). Han Dynasty Camel-Button Bronze Seal. *Natl. Palace Mus. Cat.*. (In Chinese)
[141] Shanghai Museum. (n.d.). Han Dynasty "Han Xiongnu Polu Zhang" Seal. *Shanghai Mus. Off. Website*. (In Chinese)
[142] Luoyang Museum. (n.d.). Tang Dynasty Three-Color Green-Glazed Camel Carrying Silk. *Luoyang Mus. Off. Website*. (In Chinese)
[143] Xiang, Y. (2025). Distant Camel Bells, Lasting Charm of the Silk Road: The Three-Color Camel and Camel-Keeper Figurine in the Henan Museum Collection. *People's Daily*, 2025-09-05. (In Chinese)
[144] Lu, Q. F., Zhang, H. L., Yan, H., & Li, J. P. A Northern Wei Tomb at Zhucang, Mengjin, Luoyang. *Cult. Relics* **2012**, (12). (In Chinese)
[145] Li, H. P., Feng, S. H., Han, Z. P., Shi, Y. T., Wang, Y. L., Ma, L. N., Zhang, Y. Y., Deng, X. B., Li, S. Q., & Li, Q. Z. Excavation of Two Northern Wei Tombs in Yanshi, Luoyang. *Cult. Relics Cent. China* **2019**, (6). (In Chinese)
[146] Zhang, Q. J., Lü, J. C., Ji, B. J., Li, B. J., Gao, S., Sun, X. T., Zhang, W. J., & Chen, Y. X. Excavation of Northern Wei Tomb M11 at Hudong, Datong County, Shanxi. *Cult. Relics* **2014**, (1). (In Chinese)
[147] Shaanxi Provincial Institute of Archaeology. Excavation of Tomb M21 at Maopo Village, Chang'an District, Xi'an, Shaanxi. *Archaeol. Cult. Relics* **2018**, (3), 3-15. (In Chinese)
[148] Henan Museum. (n.d.). Tricolour Camel and Its Groom. *Henan Mus. Off. Website*. (In Chinese)

[149] National Museum of History, Taiwan. (n.d.). Tang Dynasty Tricolour Camel. *Natl. Mus. Hist. Off. Website*. (In Chinese)
[150] Zhang, J. N. Appreciation of the Painted Camel Figurine Unearthed from the Tang Dynasty Mu Tai Tomb in the Collection of Qingcheng County Museum. *Identif. Apprais. Cult. Relics* **2020**, (4), 4-7. (In Chinese)
[151] Shanxi Provincial Institute of Archaeology. (2017). *The Sui Dynasty Tomb of Hulü Che at Shagou, Taiyuan*. Beijing: Science Press, 2017. (In Chinese)
[152] Shaanxi Provincial Institute of Archaeology. (2023). Archaeological Excavation Results of the Northern Zhou Tomb of Yuwen Jue in Xianyang, Shaanxi. *People's Daily Online*, 2023-09-19. (In Chinese)
[153] Lu, S. L. (n.d.). Pottery Camel Unearthed from the Sui Dynasty Tomb of Lü Sili, Xi'an (In Chinese).
[154] Emperor Qinshihuang's Mausoleum Site Museum, Xi'an Institute of Cultural Relics Preservation, Shaanxi Normal University, et al. Preliminary Research on Camel-Themed Artifacts from the Eastern Zhou to Qin-Han Periods. *Archaeol. Cult. Relics* **2025**. (In Chinese)
[155] Tibet Administrative Commission of Cultural Heritage. (1987). Investigation of the Remudong Petroglyphs in Rutog County, Tibet. Cult. Relics 1987, (3), 45-51. (Includes data on Lurilangka). (In Chinese)
[156] Bao, S. G., & Zhao, T. T. A Study of Camel Remains from the Bronze Age to Early Iron Age in the Eurasian Steppe. Agric. Archaeol. 2022, (6), 141-152. (In Chinese)
[157] Li, C. L. Analysis of the Sources and Uses of Camels in the Liao Dynasty. *J. Chifeng Univ.* **2021**, (1), 42-44. (In Chinese)
[158] Luoyang Municipal Cultural Relics Bureau. (2025). Modern Camel Caravan Retraces Silk Road, Resembling Tang Dynasty Murals Unearthed in Luoyang. *Luoyang Munic. Cult. Relics Bur. Off. Website* **2025**. (In Chinese)
[159] Zhang, J. W. (2023). Treasures of the Museum: How Does the Mural of a Hu Man Leading a Camel Laden with Silk Reflect Cultural Exchanges? *China News Service*, 2023-12-07. (In Chinese)
[160] Shandan County Museum. (2024). Molded Brick with Image of a Hu Merchant Leading a Camel, Tang Dynasty. *Shandan Cty. Mus. Off. Website* **2024**. (In Chinese)
[161] Dunhuang Museum. (2020). Tang Dynasty Molded Brick with Image of a Hu Man Leading a Camel (Left Orientation). *Dunhuang Mus. Off. Website* **2020**. (In Chinese)
[162] Zhao, Z. Y. (2026). Warm Glaze, Silk Road Imprint. *Shaanxi Daily*, 2026-02-21. (In Chinese)
[163] Shaanxi Provincial Institute of Archaeology. (2018). Newly Unearthed Stone Carvings Including 27 Ostrich Statues at Tang Yuanling Mausoleum. *China News Service*, 2018-02-08. (In Chinese)
[164] China Times News. (2026). Archaeological Revelation: Restoration of Tang Dynasty Gilt Bronze Armor in Qinghai Reveals Tuyuhun Royal Style. *China Times News*, 2026-01-15. (In Chinese)
[165] Luoyang Municipal Institute of Cultural Relics and Archaeology. Excavation of a Tang Dynasty Three-Color Glazed Tomb (C7M5657) at Guanlin, Luoyang, Henan. *Cult. Relics* **2020**, (2). (In Chinese)

[166] Shanxi Provincial Institute of Archaeology. (2024). Archaeological Data Released on the Tang Dynasty Mural Tomb at Luanshitan, Taiyuan. *Guangming Daily*, 2024-06-06. (In Chinese)
[167] Dunhuang Museum. Tang Dynasty Moulded Brick Tombs at Foyemiaowan, Dunhuang (Part IV). *Dunhuang Res.* **2018**, (3), 22-29. (In Chinese)
[168] Tianshui Museum. Excavation of a Tang Dynasty Tomb at Shimaping, Tianshui City, Gansu. *Archaeol. Cult. Relics* **1999**, (2), 32-36. (In Chinese)
[169] Baoji Archaeological Team. Excavation of a Song Dynasty Tomb at Changling Machinery Factory in Baoji, Shaanxi. *Archaeol. Cult. Relics* **1998**, (6), 23-28. (In Chinese)
[170] Qingyang Museum. Brief Report on the Tomb of the Fifth Year of the Xuanhe Era of the Northern Song Dynasty in Zhenyuan, Gansu. *Longyou Wenbo* **2001**, (2), 15-19. (In Chinese)
[171] Henan Provincial Museum. Excavation of a Jin Dynasty Tomb in Jiaozuo, Henan. *Cult. Relics* **1979**, (8), 12-19. (In Chinese)
[172] Chengdu Institute of Cultural Relics and Archaeology. (2004). Excavation of a Song Dynasty Tomb at Zhihe Village, Qinglong Township, Chengdu. In *Archaeological Discoveries in Chengdu, 2002*. Beijing: Science Press, 2004, pp. 156-163. (In Chinese)
[173] Zheng, L. Excavation of a Western Xia Tomb at Dashata, Jungar Banner. *Inner Mongolia Cult. Relics Archaeol.* **1981**, (Inaugural Issue), 34-39. (In Chinese)
[174] Nanjing Museum. (1981). *The Ming Xiaoling Mausoleum*. Beijing: Cultural Relics Press, 1981, pp. 45-47. (In Chinese)
[175] Hu, H. S. (2013). *A Study of the Ming Thirteen Tombs*. Beijing: Beijing Yanshan Press, 2013, pp. 78-82. (In Chinese)
[176] Beijing Municipal Institute of Cultural Relics. Investigation of the Jingtai Tomb of the Ming Thirteen Tombs. *Beijing Wenbo* **2003**, (4), 23-28. (In Chinese)
[177] Li, D. Q. A Study of the Xianling Mausoleum of the Ming Dynasty. *Jianghan Archaeol.* **1990**, (2), 56-62. (In Chinese)
[178] Nanjing Municipal Museum. The Tombs of Ming Princes Xu Da, Li Wenzhong, and Mu Ying. *Nanjing Cult. Relics* **1996**, (2), 34-39. (In Chinese)
[179] Anhui Provincial Institute of Cultural Relics and Archaeology. (2001). *The Ming Imperial Mausoleum*. Beijing: Cultural Relics Press, 2001, pp. 67-70. (In Chinese)
[180] Xiangfan Municipal Office for the Preservation of Cultural Relics. The Tomb of Prince Xiangjian of the Ming Dynasty in Xiangyang. *Jianghan Archaeol.* **1983**, (2), 41-46. (In Chinese)
[181] Henan Provincial Institute of Cultural Relics and Archaeology. (2006). *The Tomb of Prince Lujian in Xinxiang*. Zhengzhou: Zhongzhou Ancient Books Publishing House, 2006, pp. 89-93. (In Chinese)
[182] Baoji Bronze Ware Museum. (2024). Silk Road Camel Bells: Northern Song Dynasty Hu People Leading Camel Pictorial Brick. *Baoji Bronze Ware Mus. Off. Website* **2024**. (In Chinese)
[183] Tang, H. S. (2001). *Qinghai Petroglyphs: A Study of Binary Opposition Thinking and Its Concepts in Prehistoric Art*. Beijing: Science Press, 2001, pp. 156-160. (In Chinese)

[184] Baotou Municipal Office for the Preservation of Cultural Relics. Investigation of Petroglyphs at Guyang, Baotou, Inner Mongolia. *Inner Mongolia Cult. Relics Archaeol.* **2019**, (2), 23-28. (In Chinese)
[185] Alxa Museum. Investigation Report on the Jiruken Zhadegai Petroglyphs in Alxa Left Banner. *Inner Mongolia Cult. Relics Archaeol.* **2020**, (3), 45-51. (In Chinese)
[186] Gai, S. L. (1989). *Ulanqab Petroglyphs*. Beijing: Cultural Relics Press, 1989, pp. 234-237. (In Chinese)
[187] Gai, S. L. (1986). *Yinshan Petroglyphs*. Beijing: Cultural Relics Press, 1986, pp. 178-182. (In Chinese)
[188] Xinjiang Institute of Cultural Relics and Archaeology. Investigation of Petroglyphs at Zheyaogou, Qincheng, Hami. *Xinjiang Cult. Relics* **1995**, (2), 34-39. (In Chinese)
[189] Xinjiang Museum. Investigation of Petroglyphs at Bostan Pasture, Mori County. *Archaeol. Cult. Relics* **1997**, (4), 23-28. (In Chinese)
[190] Jiayuguan Municipal Office for the Preservation of Cultural Relics. (2003). *Heishan Petroglyphs in Jiayuguan*. Lanzhou: Gansu People's Publishing House, 2003, pp. 89-92. (In Chinese)
[191] Gansu Provincial Museum. Investigation of Petroglyphs at Daheigou, Subei Mongol Autonomous County. *Archaeology* **1994**, (7), 56-61. (In Chinese)
[192] Xu, X. G. Investigation of the Halong Petroglyphs in Gangca County, Qinghai. *Qinghai Cult. Relics* **1990**, (4), 12-18. (In Chinese)
[193] Tang, H. S. Investigation and Research on the Lushan Petroglyphs. *Acta Archaeol. Sin.* **1998**, (2), 145-168. (In Chinese)
[194] Helanshan Rock Art Conservation Center. (2004). *Helankou Petroglyphs in the Helan Mountains*. Beijing: Cultural Relics Press, 2004, pp. 267-270. (In Chinese)
[195] Zhou, X. H. (2000). *Damai Petroglyphs in Zhongwei*. Yinchuan: Ningxia People's Publishing House, 2000, pp. 189-193. (In Chinese)
[196] Tibet Administrative Commission of Cultural Heritage. Investigation of the Remudong Petroglyphs in Rutog County, Tibet. *Cult. Relics* **1987**, (3), 45-51. (In Chinese)
