# Supplementary figures and images for: Following Camels Between Bone and Culture: Camel–Human Interactions in China from the Neolithic to the Late Imperial Period
Source: Animals (Basel). 2026 Mar 1;16(5):772. doi: 10.3390/ani16050772 (PMC12984582; doi:10.3390/ani16050772)

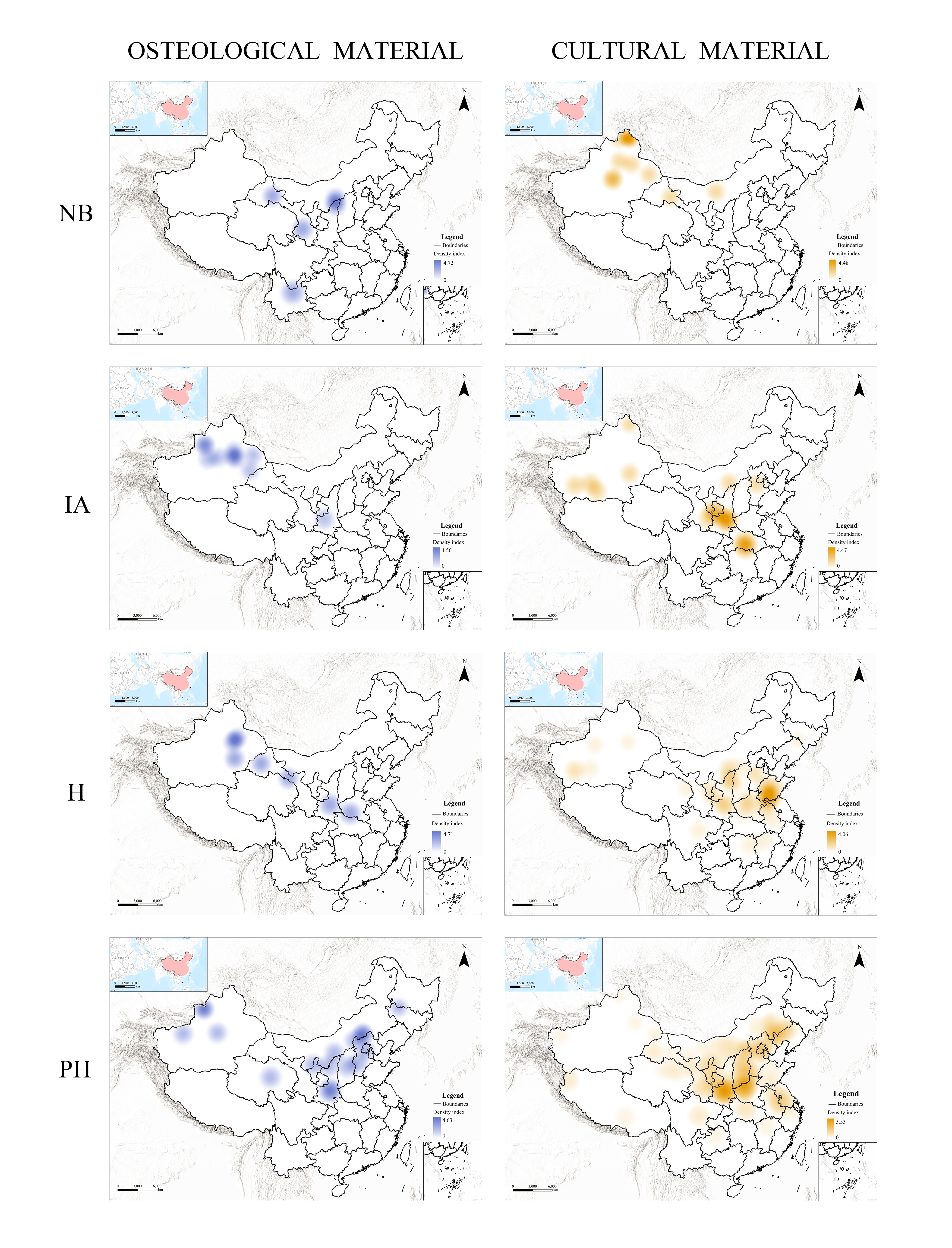

Supplement: Supplementary file 1 [file animals-16-00772-s001.zip › animals-4158267-supplementary/Figure S2.png]
